# Transcriptional organization, regulation and functional analysis of *flhF* and *fleN* in *Pseudomonas putida*

**DOI:** 10.1371/journal.pone.0214166

**Published:** 2019-03-19

**Authors:** Blanca Navarrete, Antonio Leal-Morales, Laura Serrano-Ron, Marina Sarrió, Alicia Jiménez-Fernández, Lorena Jiménez-Díaz, Aroa López-Sánchez, Fernando Govantes

**Affiliations:** 1 Centro Andaluz de Biología del Desarrollo, Universidad Pablo de Olavide/Consejo Superior de Investigaciones Científicas/Junta de Andalucía, Sevilla, Spain; 2 Departamento de Biología Molecular e Ingeniería Bioquímica, Universidad Pablo de Olavide, Sevilla, Spain; Universite Paris-Sud, FRANCE

## Abstract

The *Pseudomonas putida flhA-flhF-fleN-fliA* cluster encodes a component of the flagellar export gate and three regulatory elements potentially involved in flagellar biogenesis and other functions. Here we show that these four genes form an operon, whose transcription is driven from the upstream P*flhA* promoter. A second promoter, P*flhF*, provides additional transcription of the three distal genes. P*flhA* and P*flhF* are σ^N^-dependent, activated by the flagellar regulator FleQ, and negatively regulated by FleN. Motility, surface adhesion and colonization defects of a transposon insertion mutant in *flhF* revealed transcriptional polarity on *fleN* and *fliA*, as the former was required for strong surface adhesion and biofilm formation, and the latter was required for flagellar synthesis. On the other hand, FlhF and FleN were necessary to attain proper flagellar location and number for a fully functional flagellar complement. FleN, along with FleQ and the second messenger c-di-GMP differentially regulated transcription of *lapA* and the *bcs* operon, encoding a large adhesion protein and cellulose synthase. FleQ positively regulated the P*lapA* promoter and activation was antagonized by FleN and c-di-GMP. P*bcsD* was negatively regulated by FleQ and FleN, and repression was antagonized by c-di-GMP. FleN promoted FleQ binding to both P*lapA* and P*bcsD in vitro*, while c-di-GMP antagonized interaction with P*bcsD* and stimulated interaction with P*lapA*. A single FleQ binding site in P*lapA* was critical to activation *in vivo*. Our results suggest that FleQ, FleN and c-di-GMP cooperate to coordinate the regulation of flagellar motility and biofilm development.

## Introduction

Bacterial life cycles in the environment are commonly characterized by the alternation of a single cell-based free-living planktonic stage and a sessile stage during which they develop structured highly cooperative surface-associated communities, also known as biofilms [1]. While the planktonic lifestyle allows bacterial cells to colonize new niches and gain access to fresh resources (and also escape from unfavourable habitats), biofilm growth has been shown to promote positive interactions between organisms, such as syntrophism and horizontal genetic transfer, while providing a nurturing, protective environment [2,3]. For many bacteria, motility during the planktonic stage is directed by flagella, molecular engines that enable swimming in liquid and semi-solid media by engaging in propeller-like rotation driven by the protonmotive force [4]. Flagella are complex organelles requiring the hierarchical synthesis and assembly of multiple structural and functional elements [5]. On the other hand, biofilm formation is generally considered a form of coordinated collective behaviour reminiscent of some developmental processes in higher organisms. Biofilm development proceeds through stages of adhesion, proliferation and microcolony formation and maturation, and is terminated by programmed biofilm dispersal [6,7]. Transition between the planktonic and biofilm lifestyles and the ordered succession of such stages arguably requires a variety of signal transduction and regulatory pathways to connect environmental and physiological signals to the adequate physiological responses [7].

*Pseudomonas putida* is a well-characterized Gram-negative soil and rhizosphere bacterium and a highly versatile model organism for biodegradation of organic toxicants, and bioremediation of polluted sites [8]. During planktonic growth, *P*. *putida* displays unipolar lophotrichous flagellation (i.e., carries a tuft of flagella at a single pole) [9]. In the reference strain KT2440 genome [10], genes encoding the structural components of the flagella and the chemotaxis signal transduction system, as well as a number of dedicated regulatory proteins, are encoded in a near-contiguous 70.7 kbp cluster containing 70 ORFs, of which at least 63 are apparently related to flagellar motility and chemotaxis. In addition, 27 genes encoding MCP chemoreceptors are scattered elsewhere in the genome [11]. The ability to form biofilms on both biotic and abiotic surfaces is a key to *P*. *putida* survival in its natural environment, and several factors relevant to biofilm development in *P*. *putida* have been identified [12]. The high molecular weight adhesin proteins LapA and LapF are important determinants for cell-surface and cell-cell interactions [13], flagella have been shown to contribute to initial surface attachment and to the maturation stage [12]. The major component of the extracellular matrix in *P*. *putida* biofilms is a mixture of exopolysaccharides (EPS), whose synthesis and export functions are encoded in four separate gene clusters in the *P*. *putida* chromosome, and the contribution of different types of EPS to the extracellular biofilm matrix and biofilm stability has been explored [14–17].

The nucleotide c-di-GMP is ubiquitously used in bacteria for intracellular signalling of the transition between the planktonic and sessile lifestyles. c-di-GMP is synthesized from GTP by diguanylate cyclase (DGC) activities, and degraded by specific phosphodiesterase (PDE) activities. Bacterial genomes often encode multiple proteins displaying one or both of these activities, implying that the c-di-GMP levels are likely regulated in a complex fashion. Changes in c-di-GMP concentration are sensed by effectors, which in turn regulate a variety of processes, generally related to motility, biofilm development or virulence, acting at the transcriptional, translational or posttranslational levels. The biology of c-di-GMP signalling has been extensively reviewed (see for example [18,19]).

FleQ has long been known as an enhancer-binding protein of the NtrC/NifA family of σ^N^-dependent promoter activators and as the master regulator of flagellar biogenesis in *Pseudomonas aeruginosa* and other bacteria [20–23]. In *P*. *aeruginosa* FleQ is a c-di-GMP-responsive transcription factor that reversely regulates genes involved in flagellar motility and surface adhesion in response to changes in the intracellular levels of this second messenger [24]. Recent work by our lab and others has also highlighted the importance of FleQ and c-di-GMP in the switch from the planktonic to the sessile lifestyle and vice versa in *P*. *putida* [23,25]. Transcription of the flagellar cluster is subjected to a regulatory cascade in which the σ^N^-dependent activator FleQ is the master regulator, c-di-GMP antagonizes FleQ activation, and the flagellar σ factor FliA directs transcription of late flagellar and chemotaxis genes. On the other hand, synthesis of key components of the biofilm matrix, such as the high molecular weight adhesin LapA or the cellulose synthase complex is subjected to FleQ- and c-di-GMP-dependent regulation to ensure maximum expression under high c-di-GMP regimes [23]. Finally, biofilm dispersal is triggered by a decrease in c-di-GMP concentration provoked by the PDE BifA [26], which is indirectly regulated by FleQ *via* the flagellar cascade, thus providing a regulatory link between the termination of the biofilm lifestyle and the synthesis of new flagella [23].

FlhF and FleN (also known as FlhG, YlxH or MinD2), two members of the SIMIBI family of nucleotide-binding proteins, have been characterized as responsible of the spatial and numerical regulation of flagellar biogenesis in a variety of bacteria showing polar flagellation [27–30], as well as the peritrichously-flagellated *Bacillus subtilis* [31]. FlhF is a Signal Recognition Particle (SRP)-type GTPase responsible of the polar localization of flagella in *Vibrio*, *Shewanella*, *Campylobacter*, *Helicobacter*, and *Pseudomonas aeruginosa* [32–37]. FleN is a MinD/ParA-type ATPase involved in restricting the number of flagella to that characteristic for each organism [31,32,38–41]. Based on biochemical, genetic and structural evidence, it has been proposed that GTP-bound FlhF initiates flagellar biogenesis by recruiting early components of the flagellar basal body to the pole, and FleN antagonizes FlhF function by stimulating its GTPase activity once the correct number of flagella (or flagellar basal bodies) is attained, but many mechanistic details of this model are as of yet unexplored [4,28,42].

In addition to their roles in determination of flagellar location and number, FlhF and FleN have been involved in transcriptional regulation of the flagellar genes in different organisms. FlhF and the flagellar type-III secretion system protein FlhA negatively regulate flagellar gene transcription in *Helicobacter pylori* [37]. FleN antagonizes FleQ-dependent activation of Class II flagellar promoters in a c-di-GMP-dependent manner in *P*. *aeruginosa*, etc [43]. In addition, FleN modulates FleQ-dependent regulation of the *pel*, *pea* and *cdrA* operons, encoding EPS components and a large adhesin, in *P*. *aeruginosa* [44], and has recently been shown to play a similar role in the *lapA* and *bcs* operons in *P*. *putida* [25].

Recently, we described the isolation of a transposon insertion mutant in *flhF* in *P*. *putida* KT2442, a rifampicin-resistant derivative of the *P*. *putida* reference strain KT2440, displaying major defects in biofilm formation and flagellar motility [45]. Here we address the origin of the phenotypic defects observed in this mutant and explore the roles of FlhF and FleN in the regulation of flagellar gene expression and flagellar biogenesis and in the transcriptional regulation of biofilm matrix components.

## Results

### The *flhAF-fleN-fliA* gene cluster is co-transcribed as an operon

**Fig 1:**
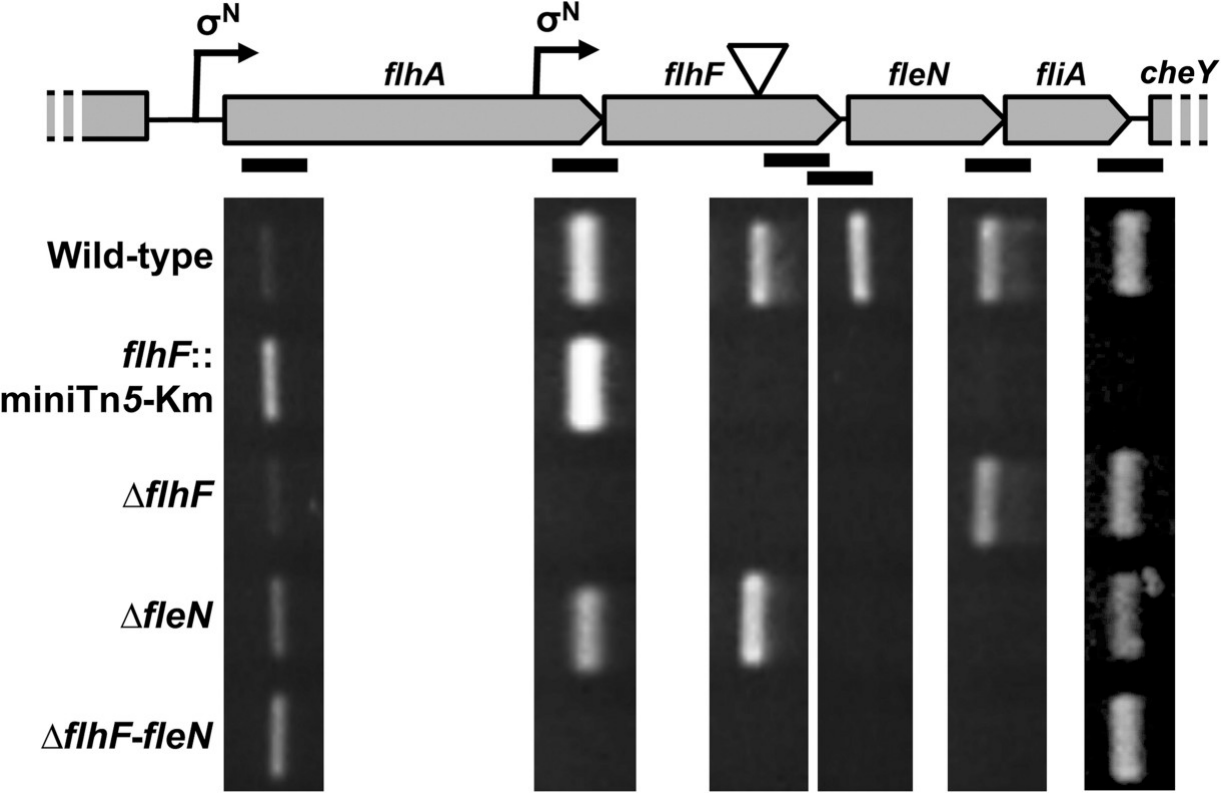
Transcriptional organization of the *flhA-flhF-fleN-fliA* cluster. (Top) Cartoon of the of the *flhA-flhF-fleN-fliA-cheY* genes showing the location of the putative σ^N^-dependent promoters (bent arrows) and the location of the transposon insertion in the *flhF*::miniTn*5*-Km mutant (Bottom) Ethidium bromide-stained agarose gel showing the results of semiquantitative RT-PCR assays using total RNA from the wild-type, *flhF*::miniTn*5*-Km. Δ*flhF*, Δ*fleN* and Δ*flhF-fleN* strains, and primers annealing to the *flhA* coding sequence, the *flhF* coding sequence (distal region), and flanking each intergenic region (black bars).

In a genetic screen for transposon mutant derivatives of *P*. *putida* KT2442 (a rifampicin-resistant derivative of the model strain KT2440) with defects in biofilm formation we recently isolated MRB49, bearing a miniTn*5-*Km insertion in the flagellar gene *flhF*. MRB49 displayed severe defects in both biofilm formation and flagellar motility that were comparable to those of a *fleQ* mutant [45], suggesting that the mutation impairs a key step in the coordinated regulation of flagellar biogenesis and surface adhesion. The gene *flhF* is the second of a string of fourteen genes (*flhAF-fleN-fliA-cheYZAB-motCD-pp4334-pp4333-cheW-pp4331*) transcribed in the same orientation. We have identified a FleQ-activated, σ^N^-dependent (i.e., Class II) promoter activity upstream from *flhA* [23]. On the other hand, Rodríguez-Herva *et al*. (2010) [46] identified *cheY* as a target for FliA regulation, suggesting that *cheY* and the genes downstream are part of a different, FliA-dependent (i.e., Class IV) transcriptional unit. However, the transcriptional organization of the *flhA-flhF-fleN-fliA* region is currently unresolved, as Pandza *et al*. (2000) [47] proposed the transcriptional units *flhA* and *flhF-fleN-fliA*, and Rodríguez-Herva *et al*. (2010) [46] proposed the transcriptional units *flhAF* and *fleN-fliA*. To solve this discrepancy, semiquantitative RT-PCR was performed using RNA from the wild-type strain KT2442 and the *flhF*::miniTn*5*-Km mutant MRB49 as templates and specific primers amplifying a proximal segment of *flhA*, the *flhA-flhF*, *flhF-fleN*, *fleN-fliA* and *fliA-cheY* intergenic regions, and a distal segment of *flhF* (downstream from the transposon insertion) (Fig 1).

RT-PCR from the wild-type strain yielded amplified products from all six primer pairs. In contrast, RNA from MRB49 (*flhF*::miniTn*5*-Km) yielded bands from the *flhA* and *flhA-flhF* segments, but no product was obtained with any of the oligonucleotide pairs annealing downstream from the transposon insertion point, indicating that the transposon insertion is polar on the downstream *fleN* and *fliA*. In disagreement with previous reports [46,47], these results rule out the presence of transcriptional terminators at the *flhA-flhF*, *flhF-fleN*, *fleN-fliA* and *fliA-cheY* junctions, indicating that *flhA*, *flhF*, *fleN*, *fliA* and *cheY* are co-transcribed as part of the same operon.

As the miniTn*5*-Km insertion in *flhF* present in MRB49 causes transcriptional polarity on *fleN* and *fliA*, we questioned whether the phenotypes displayed by the *flhF*::miniTn*5*-Km mutant may be due to the defect in expression of one of these genes, rather than the inactivation of *flhF*. To test this hypothesis, we used allelic replacement to construct KT2442-derived mutant strains MRB69, MRB71 and MRB78, bearing unmarked complete deletions of *flhF*, *fleN* and both genes simultaneously (see Materials and Methods below). RNA from MRB69, MRB71 and MRB78 was also used as template for RT-PCR, confirming that each mutant failed to transcribe the deleted gene(s), while expression of the downstream genes was unaffected (Fig 1).

Examination of the RT-PCR results provided two additional pieces of information regarding the expression of the *flhAF-fleN-fliA* operon. Firstly, the signal corresponding to the proximal segment of *flhA* was increased in all three mutants not expressing FleN relative to the wild-type and Δ*flhF* mutant. Notwithstanding the low sensitivity of this technique for quantitative analysis, this result hints to a possible negative effect of FleN on transcription from the P*flhA* promoter. Secondly, all amplified products downstream from *flhA* were more abundant than the corresponding products obtained with the proximal *flhA* primers in every genetic background, suggesting that a second promoter activity may contribute to increase the expression of *flhF*, *fleN* and *fliA*.

### Two FleQ- and FleN-regulated promoters control *flhAF-fleN-fliA* transcription

Inspection of the sequences upstream from *flhF* and *flhA* revealed two regions displaying high similarity to the σ^N^ binding motif consensus (TGGCACG-N_4_-TTGCW) [48]. The first of these regions, bearing the sequence TGGAAAGcttcTTGCA (matches to the consensus underlined), is located between positions -71 and -57 relative to the *flhA* start codon, and likely corresponds to the promoter activity we previously designated P*flhA* [23]. The second region, bearing the sequence TGGAACAgattTTGCT (matches to the consensus underlined), is located within the *flhA* coding region, between positions -298 and -284 relative to the *flhF* start codon, and corresponds to the P1 promoter previously identified in this region [49]. We have designated this putative σ^N^-dependent promoter P*flhF* (Fig 1).

**Fig 2:**
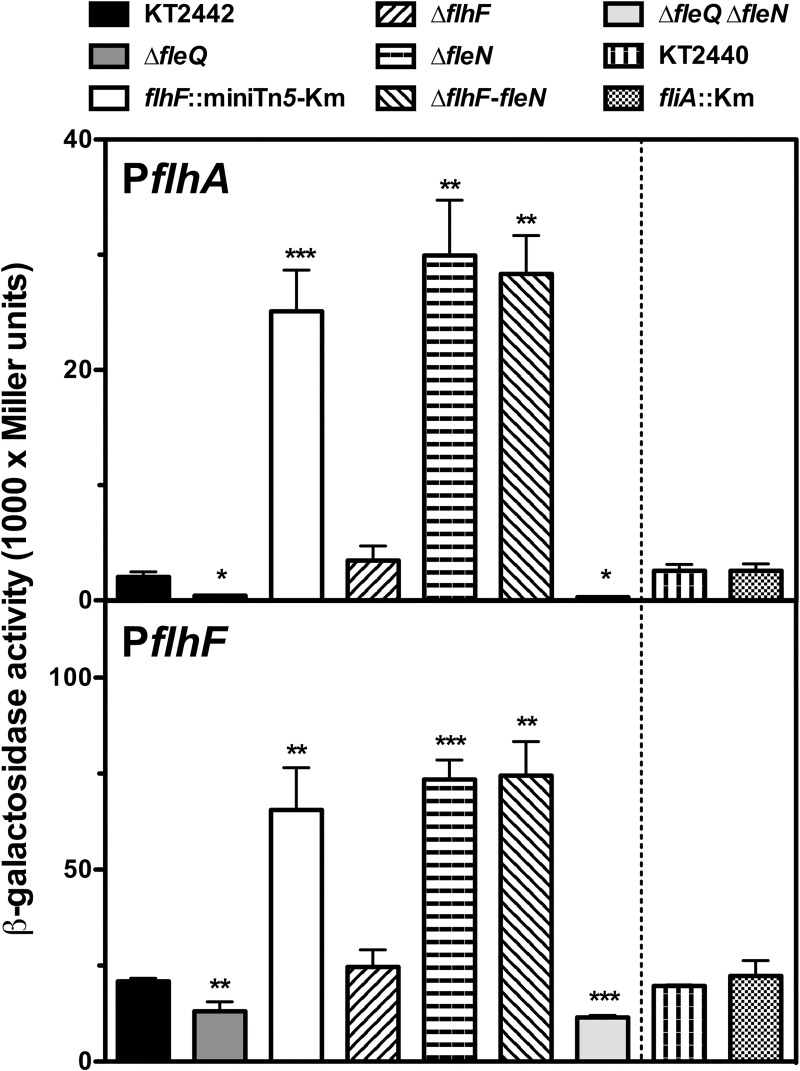
Expression of the P*flhA* and P*flhF* promoters in *P*. *putida*. β-galactosidase assays of the P*flhA* and P*flhF* promoter fusions in wild-type (KT2442), Δ*fleQ* (MRB52), *flhF*:miniTn*5*-km (MRB49), Δ*flhF* (MRB69), Δ*fleN* (MRB71), Δ*flhF-fleN* (MRB78), and Δ*fleQ*Δ*fleN* (MRB101) backgrounds (left panels) and wild type (KT2440) and *fliA*^-^ backgrounds (right panels). Bars represent the averages and standard deviations of at least three independent assays. Stars designate p-values for the Student's t-test for unpaired samples not assuming equal variance. *: p<0.05; **: p<0.01; ***:p<0.005.

To evaluate the transcription from these promoters we used plasmids pMRB115 and pMRB158, bearing transcriptional fusions of P*flhA* and P*flhF* in the broad host-range dual *gfp-lacZ* reporter plasmid pMRB3 [23]. The resulting P*flhA-gfp-lacZ* and P*flhF-gfp-lacZ* fusion plasmids were transferred to *P*. *putida* KT2442 and its Δ*fleQ*, *flhF*::miniTn*5*-Km, Δ*flhF*, Δ*fleN* and Δ*flhF-fleN* mutant derivatives by mating, and expression was assessed by means of β-galactosidase assays (Fig 2).

Expression from the P*flhA* promoter was all but abolished in the absence of FleQ relative to that in the wild-type strain, as expected from the fact that P*flhA* is a Class II flagellar promoter [23]. Consistently with the RT-PCR results above, P*flhA* transcription was increased 12- to 15-fold in the three backgrounds not expressing FleN (*flhF*::miniTn*5*-Km, Δ*fleN* and Δ*flhF-fleN*). In contrast, expression in the Δ*flhF* mutant was not significantly different from that in KT2442. The β-galactosidase activity levels from the P*flhA-gfp-lacZ* fusion were also determined in the *fliA* mutant KT2440*fliA*::*aph-3* and its parent strain, KT2440. Expression in both strains was indistinguishable from that in KT2442. The putative P*flhF* promoter showed similarly high expression levels in the wild-type KT2442 and KT2440 strains, indicating that a promoter activity is indeed present in the distal region of *flhA*. The regulatory pattern of P*flhF* was very similar to that observed with P*flhA*, but the extent of the regulation was smaller in P*flhF*. Accordingly, P*flhF* expression was decreased 2-fold in the Δ*fleQ* mutant and increased 3- to 4-fold in the *flhF*::miniTn*5*-Km, Δ*fleN* and Δ*flhF-fleN* strains, relative to the wild-type. No significant differences in β-galactosidase levels were observed with the Δ*flhF* and *fliA* mutants. Taken together, these results indicate that both P*flhA* and P*flhF* are positively regulated by FleQ and negatively regulated by FleN. P*flhA* and P*flhF* expression was also assessed in the double Δ*fleQ*Δ*fleN* mutant derivative of KT2442 MRB101 (Fig 2). Expression was in both cases low and similar to that obtained in a Δ*fleQ* mutant, suggesting that FleN-dependent regulation operates *via* FleQ in both promoters. The β-galactosidase activity values and the intensities of the amplified RT-PCR fragments suggest that P*flhA* is a weaker, more stringently regulated promoter, while P*flhF* is a stronger, moderately regulated promoter. We propose that P*flhF* is responsible for most of the transcription of *flhF*, *fleN* and *fliA*, while P*flhA* drives *flhA* transcription and has a minor contribution to the expression of the three distal genes. Moreover, it is worth noting that, unlike its *P*. *aeruginosa* counterpart [21], *P*. *putida fliA* transcription is positively regulated by FleQ. Kim *et al*. (1995) [49] described the regulation of P*flhF*, and the presence of additional, σ^70^-dependent promoters that may be accountable for the high basal levels.

### FlhF and FleN are required for fully functional lophotrichous flagellation

**Fig 3:**
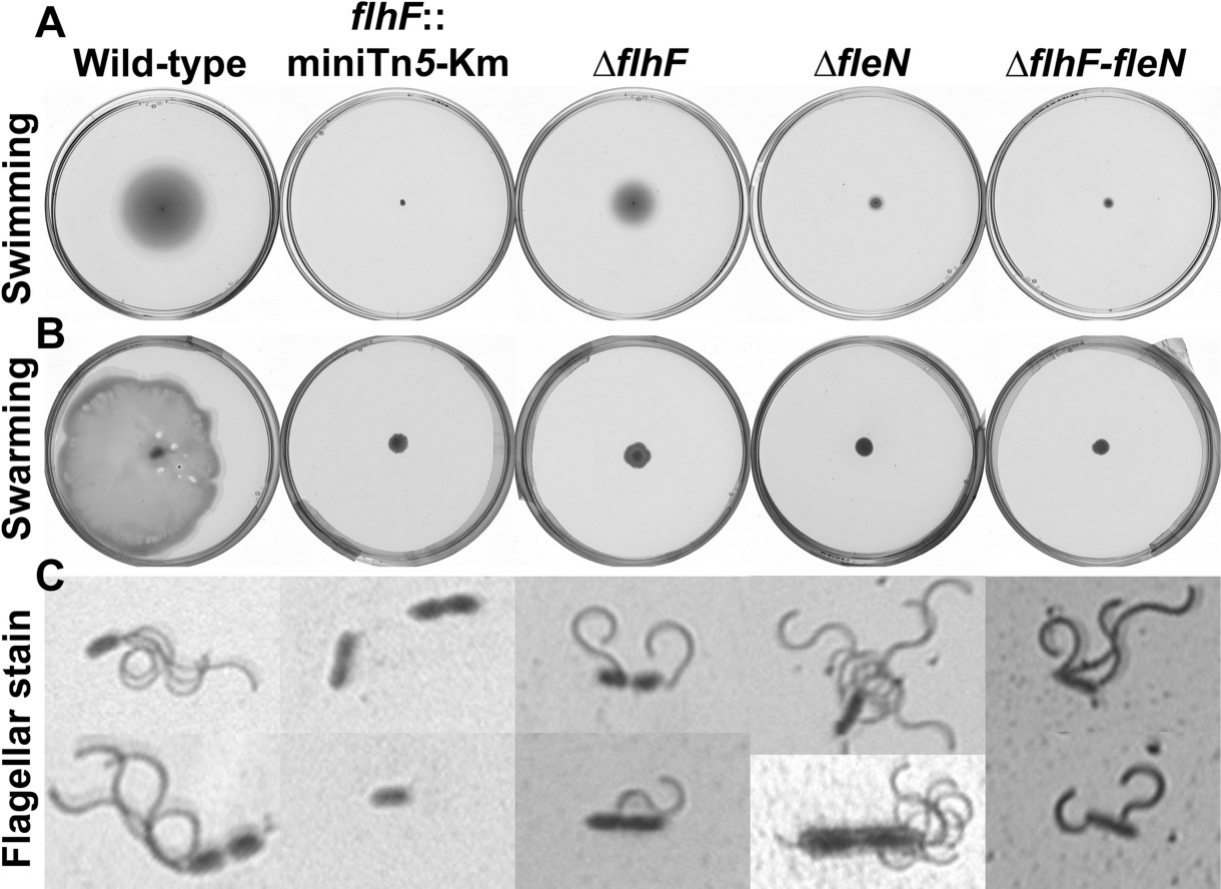
Flagellar motility of the *flhF* and *fleN* mutants. (A) Swimming motility assays, (B) swarming motility assays and (C) flagellar stain of the wild-type KT2442, the *flhF*:miniTn5-km mutant MRB49, the Δ*flhF* mutant MRB69, the Δ*fleN* mutant MRB71 and the Δ*flhF-fleN* mutant MRB78 strains. Each picture is a representative one out of at least three biological replicates.

The *flhF*::miniTn*5*-Km, Δ*flh*, Δ*fleN* and Δ*flhF-fleN* mutants were tested, along with the wild-type strain KT2442, for a variety of phenotypes related to flagellar motility. The *flhF*::miniTn*5*-Km mutant was non-flagellated and non-motile, as judged by light microscopy observation of flagellar stains and swimming and swarming motility assays (Fig 2A, 2B and 2C). In contrast, the Δ*flhF*, Δ*fleN* and Δ*flhF-fleN* mutants were flagellated and motile, thus indicating that neither of these two genes is absolutely required for the biogenesis of rotating flagella. However, the deletion mutants showed alterations in flagellar number and location (Fig 3C and S1 Fig). Thus, while the wild-type strain displayed a tuft of 3–4 polar flagella, the Δ*flhF* mutant cells displayed one or rarely two flagella, often located at a subpolar or lateral position, consistent with a role of FlhF in determining the point of flagellar insertion in one of the cell poles. The Δ*fleN* mutant displayed a bundle of flagella in a polar position, but the number of flagella (4–7) was on average greater than that in the wild-type. Finally, the Δ*flhF-fleN* mutant MRB78 showed a combination of the phenotypes of the single deletion mutants, as it displayed several flagella (2–4) inserted at polar, subpolar or lateral positions on the cell surface. All three deletion mutants were defective in swimming motility (Fig 3A): the Δ*flhF* mutant displayed a 57±1% decrease in the diameter of the swimming halo, and the corresponding values for the Δ*fleN* and the double Δ*flhF-fleN* mutant were 82±1% and 89±1%, respectively. Finally, swarming motility was undetectable in all three deletion mutants. These results indicate that even though all three deletion mutants are flagellated and motile, the observed changes in flagellar number and location point likely result in a diminished ability to coordinate flagellar rotation to achieve efficient swimming and swarming.

To investigate further the observed motility phenotypes, light microscopy-coupled video imaging was used to assess the near-surface motility patterns of the wild-type, Δ*flhF*, Δ*fleN* and Δ*flhF-fleN* strains (S2 Fig and S1–S4 Movies). Motile cells of the wild-type strain displayed a characteristic swimming pattern in which smooth and fast directional runs alternated with abrupt stops often followed by periods of slower, erratic swimming with frequent changes in direction (S2 Fig and S1 Movie). Long runs were rarely observed in motile cells of the Δ*flhF* mutant, erratic motility was highly prevalent in this organism, and cells were often propelled or tumble sideways or diagonally (S2 Fig and S2 Movie). Motile cells of the Δ*fleN* mutant showed faster near-surface motility than wild-type cells. Stops and direction changes were less frequent than in the wild-type, and uninterrupted runs often showed a tendency to bend in the clockwise direction to draw near-circular trajectories (S2 Fig and S3 Movie). The behaviour of the Δ*flhF-fleN* mutant combined fast long runs with an exacerbated tendency to clockwise-bent trajectories, along with cells displaying a "tumbling" sideways or diagonal motility pattern (S2 Fig and S4 Movie). Taken together, these results indicate that the swimming and swarming defects observed in the Δ*flhF*, Δ*fleN* and Δ*flhF-fleN* mutants are not due an inability of the flagellar apparatus to propel the cells, but rather to poor coordination of the flagellar apparatus that results in unusual motility patterns, such as "tumbling", infrequent stops and direction changes, or curved swimming trajectories.

The phenotypes of the single Δ*flhF*, Δ*fleN* or the double Δ*flhF-fleN* mutants do not suffice to explain the complete lack of flagella and swimming motility observed with the *flhF*::miniTn*5*-Km mutant. However, we have shown above that the transposon insertion is polar on *fliA* transcription, while the deletion mutants display wild-type levels of *fliA* mRNA (Fig 1). A null *fliA* mutant derivative of *P*. *putida* KT2440 was previously characterized and found to be non-flagellated and non-motile [46]. Accordingly, we propose that the lack of FliA due to transcriptional polarity is the cause of the non-flagellated, non-motile phenotypes of the *flhF*::miniTn*5*-Km mutant.

### FleN, but not FlhF, is required for normal biofilm formation

**Fig 4:**
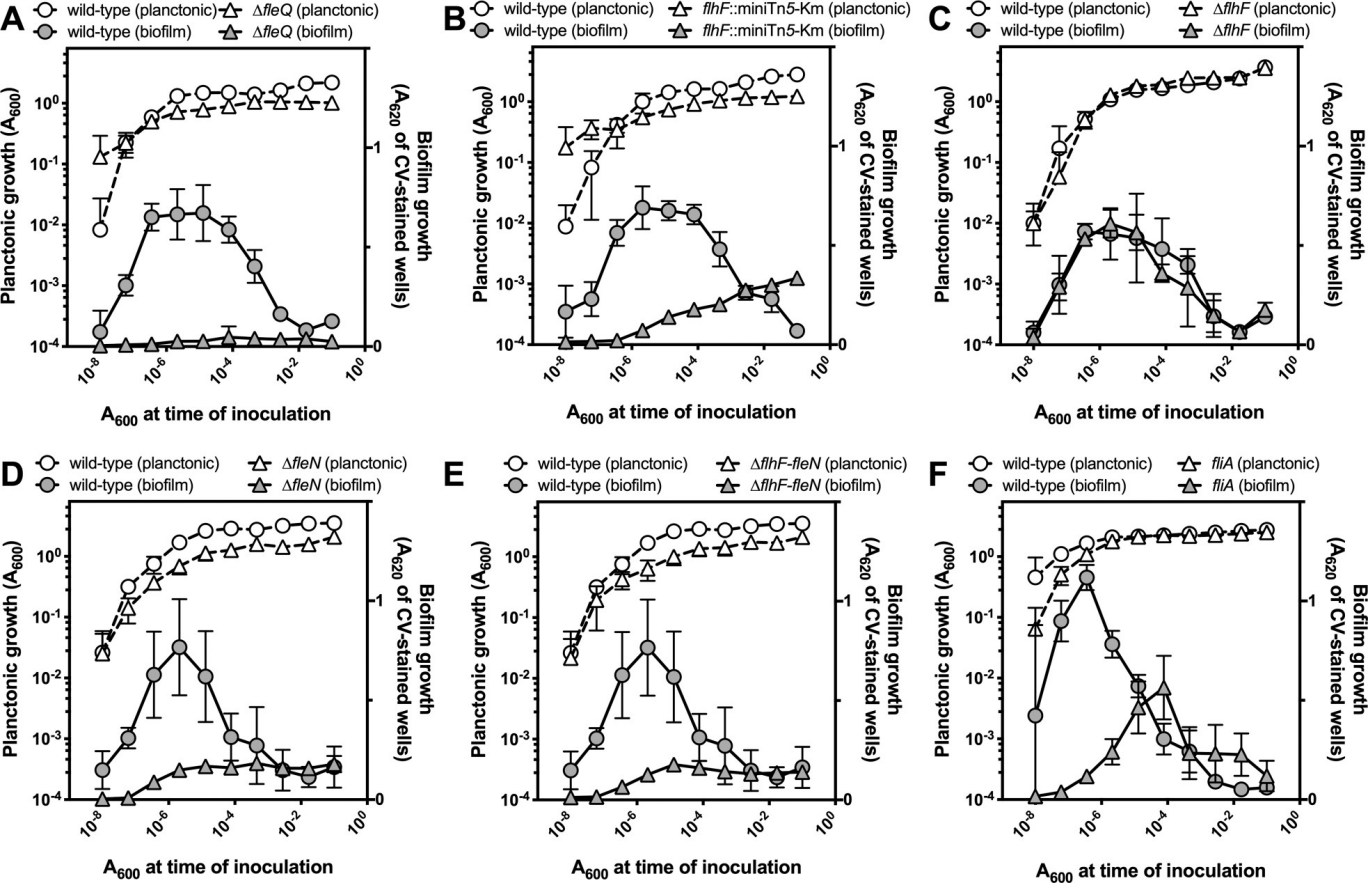
Serial dilution-based growth curves of *flhF*, *fleN* and *fliA* mutants. Planktonic (left axes, open symbols) or biofilm growth (right axes, closed symbols) is plotted against the initial A600 of each dilution. Circles represent the wild-type strain and squares represent the Δ*fleQ* mutant MRB52 (A), the *flhF*:miniTn5-km mutant MRB49 (B), the Δ*flhF* mutant MRB69 (C) the Δ*fleN* mutant MRB71 (D), the Δ*flhF-fleN* mutant MRB78 (E) or the *fliA*^-^ mutant KT2440 *fliA*::*aphA-3* (F). Plots display one representative experiment of at least three biological replicates. Error bars represent the standard deviation of the six technical replicates.

Biofilm formation by the *flhF*::miniTn*5*-Km, Δ*flhF*, Δ*fleN* and Δ*flhF-fleN* mutants was assessed by means of serial dilution-based growth curves. This method uses a dilution set and a single incubation time to recapitulate the time-course of planktonic and biofilm growth in microtiter plate wells [50]. The wild-type strain KT2442 and the Δ*fleQ* mutant MRB52 were used as positive and negative controls, respectively. The Δ*fleQ* mutant did not display any biofilm formation (Fig 4A), consistent with the phenotype documented for a *fleQ*::miniTn*5*-Km insertion mutant [45]. As previously shown [45], the *flhF*::miniTn*5*-Km mutant was impaired in biofilm formation (Fig 4B), albeit the phenotype was not so severe as in the Δ*fleQ* mutant. A similar phenotype was observed with the Δ*fleN* and Δ*flhF-fleN* mutants (Fig 4D and 4E), while the Δ*flhF* mutant displayed a biofilm growth and dispersal pattern similar to that of the wild-type strain (Fig 4B). Since the *flhF*::miniTn*5*-Km insertion is also polar on *fliA* and inactivation of *fliA* was previously proposed to impair surface attachment and biofilm formation [46], we also assessed planktonic and biofilm growth of KT2440 *fliA*::*aphA-3*, a *fliA* mutant derivative *P*. *putida* KT2442 parent strain, KT2440. As expected, the wild-type control, KT2440, displayed a planktonic and biofilm growth behavior similar to that of KT2442. The *fliA* mutant showed delayed biofilm formation and dispersal, and somewhat decreased levels of biofilm biomass (Fig 4E), a phenotype resembling that observed in a plethora of flagella-defective mutants [45], but clearly distinct and less severe than those displayed by the *flhF*::miniTn*5*-Km, Δ*fleN* and Δ*flhFfleN* mutants. Interestingly, the single common trait of these three mutants is that they do not express *fleN*. Therefore, these results strongly suggest that FleN is required for biofilm formation, while FlhF and FliA are not major players in the regulation of biofilm development.

**Fig 5:**
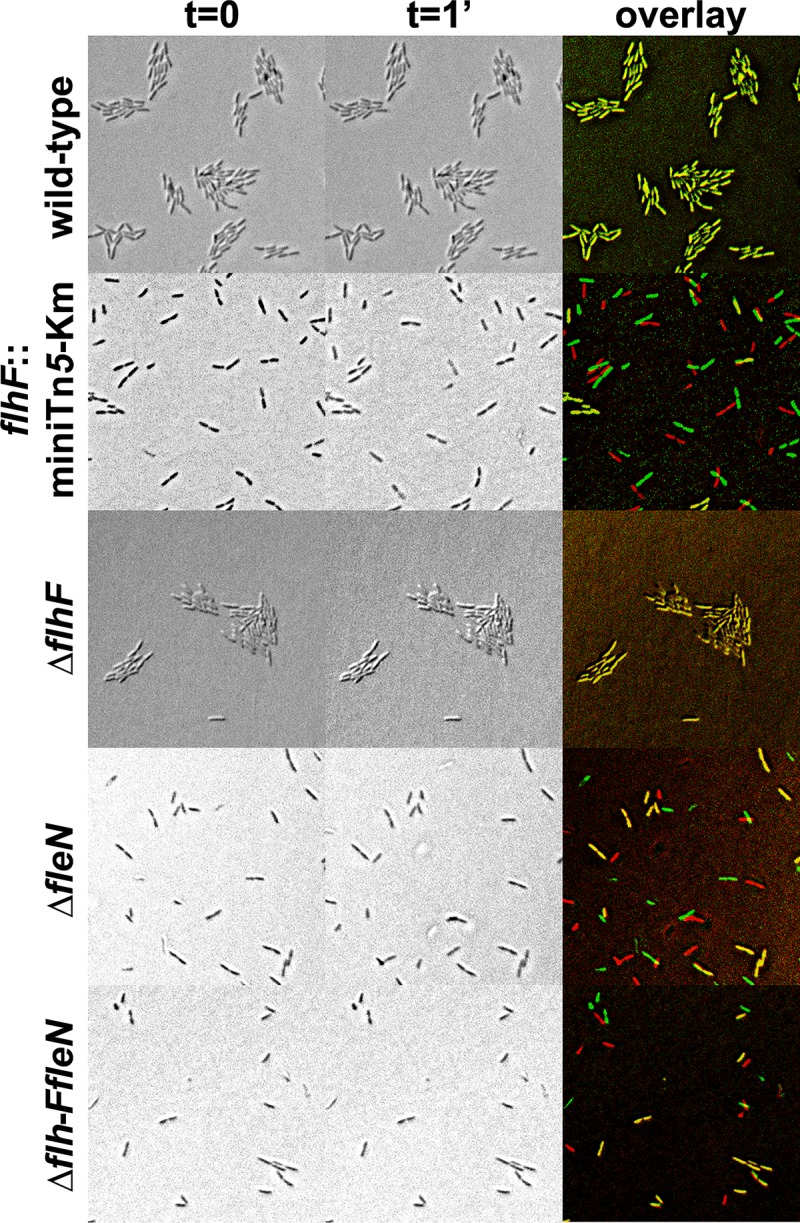
Microscopy adhesion assay of *flhF* and *fleN* mutants. Phase-contrast micrographs of (from top to bottom) the wild-type strain KT2442, the *flhF*:miniTn*5*-km mutant MRB49, the Δ*flhF* mutant MRB69, the Δ*fleN* mutant MRB71 and the Δ*flhF-fleN* mutant MRB78. Two frames of the same field were taken in a 1-minute interval (left and center). The right panel shows an overlay of the two images of each strain digitally colored red (t = 0) or green (t = 1 min).

We also assessed the impact of these mutations on surface adhesion by means of a simple attachment assay. Cells of the wild-type strain and the *flhF*::miniTn*5*-Km, Δ*flhF*, Δ*fleN* and Δ*flhF-fleN* mutants were incubated for three hours on the surface of polystyrene microtiter plate wells, the plates were placed under phase-contrast microscopy and attachment was determined by means of pairs of micrographs taken with one minute time interval. Attachment was evaluated from the brownian motility of the cells and their ability to form microcolonies (Fig 5).

Analysis of the microscopic images confirmed that the wild-type strain attaches strongly to the substrate. Attached cells were immobilized on the surface and formed microcolonies, likely due to successive divisions of substrate-attached cells. Similarly, the Δ*flhF* mutant also displayed immobilized cells in microcolonies, suggesting that the lack of FlhF does not impair surface attachment. In contrast, the strains not producing FleN (i.e., the *flhF*::miniTn*5*-Km, Δ*fleN* and Δ*flhF-fleN* mutants), incubated under the same conditions displayed a mixture of moving and immobilized cells that were seldom grouped in microcolonies. These results suggest that the lack of FleN impairs permanent attachment required for stable surface colonization.

### FleN regulates the synthesis of biofilm matrix components LapA and cellulose

**Fig 6:**
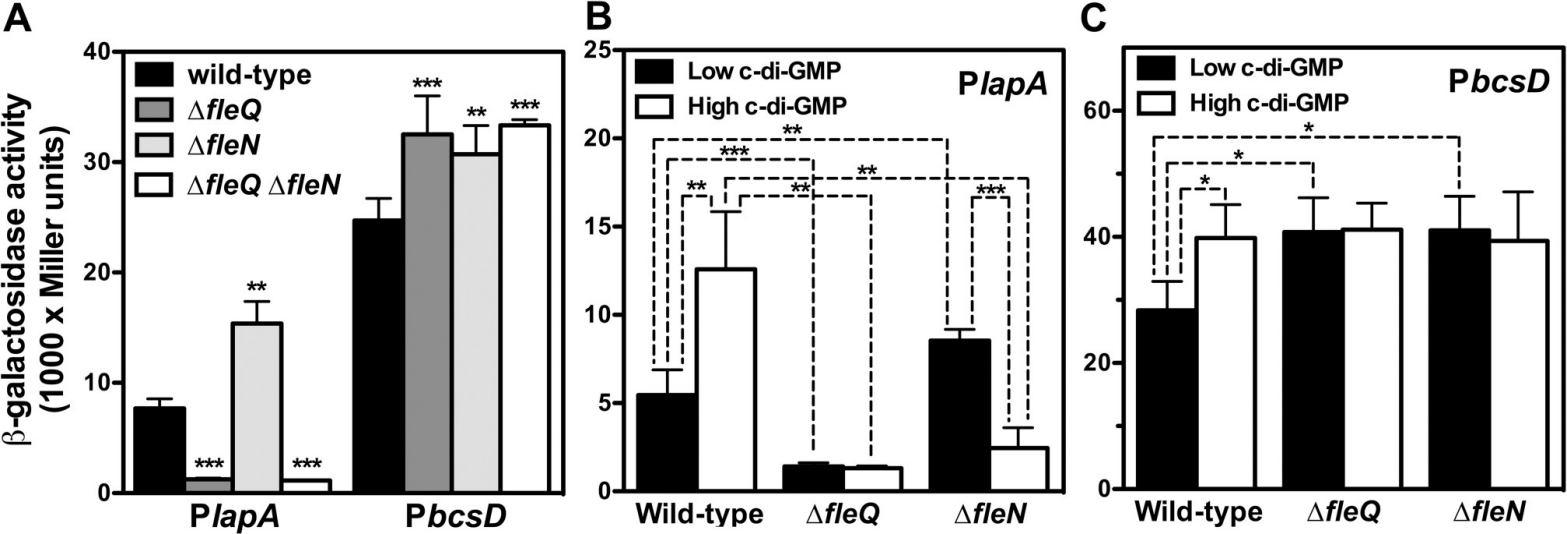
Expression of the P*lapA* and P*bcsD* promoters in *P*. *putida*. β-galactosidase assays of the P*lapA* and P*bcsD* promoter fusions in the wild-type KT2442, Δ*fleQ* (MRB52), Δ*fleN* (MRB71) and Δ*fleQ*Δ*fleN* (MRB101) backgrounds (A). PlapA (B) and PbcsD (C) promoter fusions in the wild-type KT2442, Δ*fleQ* (MRB52), Δ*fleN* (MRB71) strains bearing the YhjH-producing plasmid pMRB89 (low c-di-GMP) or the YedQ- producing plasmid pYedQ (high c-di-GMP). Bars represent the averages and standard deviations of three independent assays. Stars designate p-values for the Student's t-test for unpaired samples not assuming equal variance. *: p<0.05, **: p<0.01; ***: p<0.001.

Expression of *lapA*, encoding the high molecular weight adhesin LapA is positively regulated by FleQ [13,23,51]. Similarly, the *bcs* operon, encoding the cellulose synthesis and export cellulose synthase complex, was recently shown to be negatively regulated by FleQ [23,51]. Since mutants devoid of FleN are defective in biofilm formation, and FleN acts as an auxiliary factor to FleQ regulation in *P*. *aeruginosa* [43,44], we questioned whether FleN may also influence the expression of the P*lapA* and P*bcsD* promoters. To test this hypothesis, we transferred the P*lapA-gfp-lacZ* and P*bcsD-gfp-lacZ* fusion plasmids pMRB67 and pMRB112 to the wild-type strain KT2442 and its Δ*fleQ*, Δ*fleN* and Δ*fleQ* Δ*fleN* derivatives MRB52, MRB71 and MRB101 by mating, and expression was assessed by means of β-galactosidase assays (Fig 6A).

As previously described [23], P*lapA* expression was decreased (6-fold) in the absence of FleQ. In contrast, the β-galactosidase levels were increased 2-fold in the Δ*fleN* background. The Δ*fleQ*Δ*fleN* mutant displayed low expression levels, similar to those in the Δ*fleQ* background. These results are consistent with the notion that, similarly to the observations with the P*flhA* and P*flhF* promoters, FleN antagonizes FleQ activation of P*lapA*. P*bcsD* expression was modestly but significantly increased (1.3- to 1.4-fold) in the Δ*fleQ*, Δ*fleN* and Δ*fleQ* Δ*fleN* backgrounds. These results indicate that FleQ and FleN are both negative regulators of the *bcs* operon.

The second messenger c-di-GMP was recently shown to modulate expression of both *lapA* and the *bcs* cluster [23,51]. In *P*. *aeruginosa*, FleQ activity is regulated by c-di-GMP levels and FleN is proposed to modulate the response of FleQ to c-di-GMP [43]. To test the possible interaction of the two regulatory factors with this second messenger, the wild-type, Δ*fleQ* and Δ*fleN* strains bearing the P*lapA-gfp-lacZ* and P*bcsD-gfp-lacZ* fusion plasmids were electroporated with plasmids pMRB89, overexpressing the *E*. *coli* PDE YhjH, and pYedQ, overexpressing the *E*. *coli* DGC YedQ, which provoke c-di-GMP depletion and overproduction, respectively, and the resulting strains were assayed for β-galactosidase activity as above.

The expression patterns of the P*lapA* and P*bcsD* promoters under the c-di-GMP depletion regime were similar to those observed in the strains with physiological c-di-GMP levels: P*lapA* was 4-fold downregulated in the Δ*fleQ* strain and slightly (1.6-fold) upregulated in the Δ*fleN* strain (Fig 6B), while P*bcsD* was 1.4-fold upregulated in both mutant backgrounds (Fig 6C). These results suggest that relatively low intracellular c-di-GMP levels are naturally present in our strains under our experimental conditions. On the other hand, c-di-GMP overproduction resulted in increased expression of P*lapA* and P*bcsA*, indicating that c-di-GMP acts as an inducer on both promoters. In these conditions, P*lapA* was downregulated 10- and 5-fold, respectively, in the Δ*fleQ* and Δ*fleN* backgrounds, indicating that, while FleQ is an activator of the P*lapA* promoter under all c-di-GMP regimes, the presence of high c-di-GMP levels causes FleN to switch from antagonizing to promoting FleQ-dependent activation. In these conditions, downregulation of the P*bcsD* promoter in Δ*fleQ* and Δ*fleN* backgrounds was not observed, thus indicating that c-di-GMP acts as an inducer of P*bcsD* by releasing FleQ- and FleN-dependent repression.

### FleN and c-di-GMP modulate the interaction of FleQ with P*lapA* and P*bcsD*

To advance in the understanding of how FleN influences the expression of the P*lapA* and P*bcsD* promoters, *P*. *putida* KT2440 FleN was overproduced heterologously in E. coli NCM631 as a N-terminal CBD-intein-FleN fusion, purified by chitin affinity chromatography, and the tag was subsequently self-cleaved to release the native FleN protein. Purity of the FleN preparation was estimated to be ~60% (S3 Fig), and ATPase activity was 2,74 ± 0,54 nmol phosphate^-1^ / h^-1^ nmol FleN^-1^. This value is similar to that measured for *Vibrio alginolyticus* FlhG and ~10-fold lower than that found in *P*. *aeruginosa* FleN [39,43]

**Fig 7:**
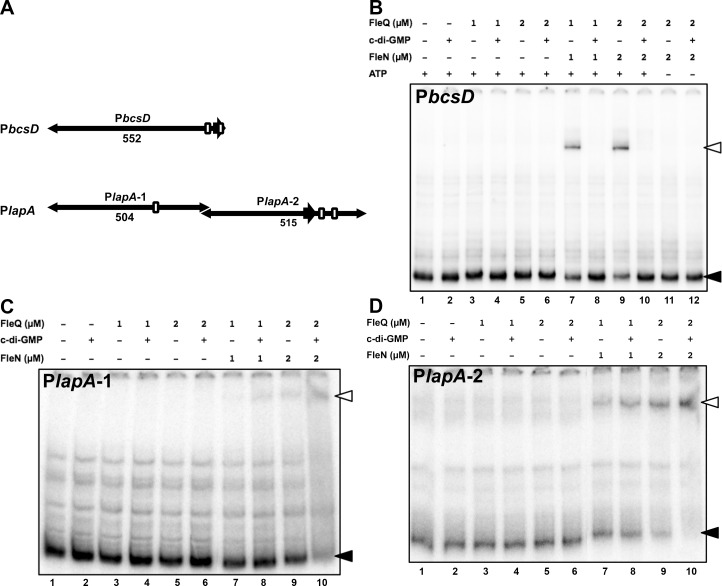
Gel mobility shift assays on the P*lapA* and P*bcsD* promoters. Panel A: Cartoon of the probes used for each of the promoter regions, indicating the sizes (in bp) of the resulting fragments and the location of the predicted FleQ binding motifs (open boxes). Drawn to scale. Panels B, C and D: Autoradiograph of a representative PAGE gel containing the indicated probe, 0, 1 or 2 μM FleQ and 0, 1 or 2 μM FleN. Assays were performed in the absence (-) or in the presence (+) of c-di-GMP and in the absence (-) or in the presence (+) of ATP. Closed arrowheads denote the free DNA probes and open arrowheads denote the retarded complexes.

Interaction of FleQ and FleN with the P*bcsD* and P*lapA* promoter regions was also assessed by means of gel mobility shift assays. To this end, three PCR products were amplified, radiolabeled and used as probes (Fig 7A): a fragment containing P*bcsD* sequences from -571 to -21 (relative to the start codon)(P*bcsD* probe), a fragment containing P*lapA* sequences from -997 to -494 (P*lapA*-1 probe), and a fragment containing P*lapA* sequences from -513 to +2 (P*lapA*-2 probe). The P*bcsD* probe is predicted to contain two FleQ binding motifs, while the P*lapA*-1 and P*lapA*-2 probes are predicted to contain one and two FleQ binding motifs, respectively [23,52] (S4 Fig). Binding reactions were performed in which each protein was added separately at concentrations of 1 or 2 μM, or together at equimolar concentrations in a binding buffer containing 50 μM ATP. In these conditions FleQ alone did not detectably retard any of the three probes (Fig 7B–7D and S5 Fig), and neither did FleN alone (S5 Fig). In contrast, the equimolar mixture of FleQ and FleN resulted in a retarded band in all three probes that was intensified as protein concentration was increased from 1 to 2 μM (Fig 7B–7D). The significantly slower relative migration rates compared with those observed previously with FleQ alone [23,51] suggests that these complexes indeed contain both proteins. We have shown above (Fig 6) that c-di-GMP acts an antagonist for FleQ repression of P*bcsD*, but as an inducer of FleQ activation at P*lapA*. Addition of c-di-GMP alone or to mixtures containing only FleQ did not alter the mobility of the probes (lanes 2, 4 and 6 in Fig 7B–7D). In contrast, a clear effect was observed upon addition of c-di-GMP to reactions containing both FleQ and FleN (lanes 8 and 10 in Fig 7B–7D), as the mobility shift of the P*bcsD* probe was completely abolished, while the retarded bands corresponding to the P*lapA*-1 and P*lapA*-2 probes were intensified. Finally, the effect of ATP on FleQ-FleN binding was assessed on the P*bcsD* promoter. In the absence of ATP, the retarded band caused by the simultaneous presence of FleQ and FleN was not observed, regardless of the presence of c-di-GMP (lanes 11 and 12 in Fig 7B). Taken together, our *in vitro* results strongly suggest that (i) FleQ directly regulates *lapA* and the *bcs* operon transcription by interacting with their promoter regions; (ii) FleQ interacts with at least one binding site at the P*bcsD* promoter region and at least two sites at the P*lapA* promoter region; (iii) FleQ binding to P*bcsD* and P*lapA* is stimulated by FleN, in a process that requires ATP (at least for P*bcsD*); and (iv) c-di-GMP acts differentially by antagonizing FleQ-FleN binding to P*bcsD* and stimulating FleQ-FleN binding to P*lapA*.

### A single FleQ binding site is critical to FleQ activation of *lapA* transcription

**Fig 8:**
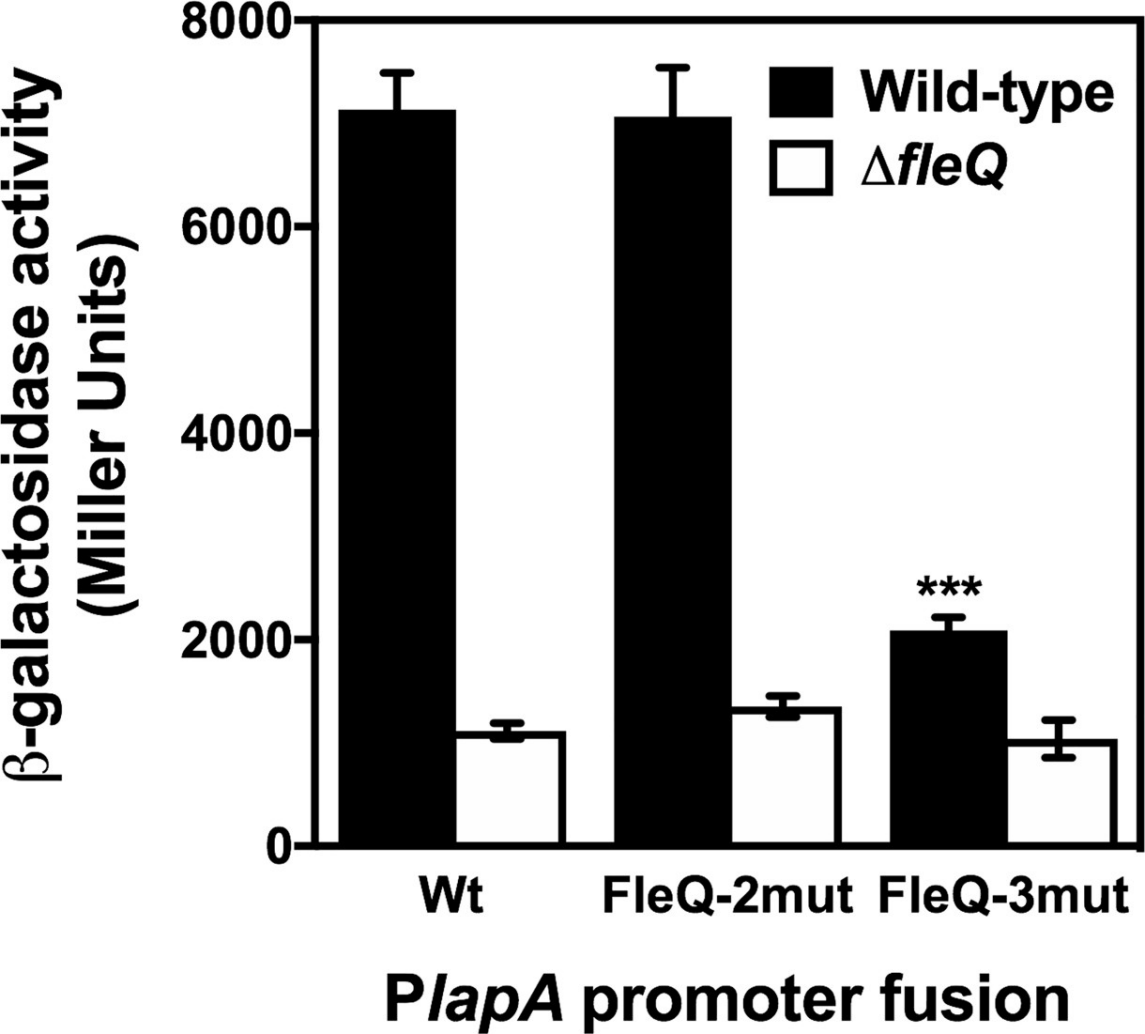
Effect of FleQ binding sites mutation on Pl*apA* expression. β-galactosidase assays of the P*lapA* promoter fusion and FleQ-2 and FleQ-3 mutated promoters fusions in the wild-type strain KT2442 and Δ*fleQ* mutant (MRB52). Bars represent the averages and standard deviations of three independent assays. Stars designate p-values for the Student's t-test for unpaired samples not assuming equal variance. *: p<0.05, **: p<0.01; ***: p<0.001.

To assess the relevance of the putative FleQ binding sites at the P*lapA* promoter region, we focused on the FleQ-2 and FleQ-3 sites, located in the vicinity of P*lapA*3, the main promoter of the six directing *lapA* transcription [52] (S2 Fig). To this end, we performed site-directed mutagenesis on the conserved residues of the FleQ-2 and FleQ-3 motifs, according to the current FleQ binding site consensus [53] (S3 Fig). Fragments containing the resulting mutant FleQ-2mut and FleQ-3mut sites were transferred to pMRB2 to yield the mutant P*lapA-gfp-lacZ* reporter plasmids pMRB80 and pMRB81, respectively. These constructs, along with the wild-type fusion plasmid pMRB67, were transferred to the wild-type and Δ*fleQ* strains, and P*lapA* expression was assessed from β-galactosidase assays (Fig 8).

Expression of the wild-type P*lapA* promoter region was high in the wild-type background, and was decreased 6-fold in the absence of FleQ, as shown above. A similar behavior was also observed with the fusion bearing the FleQ-2mut mutant motif, suggesting that, despite its proximity to the P*lapA*3 promoter, this site is not highly relevant to *lapA* transcriptional regulation. In contrast, introduction of the FleQ-3mut mutation provoked a >3-fold decrease in expression in the wild-type background, while the β-galactosidase levels were essentially unaltered in the Δ*fleQ* mutant. Still, a residual 2-fold FleQ-dependent activation was observed. These results strongly suggest that FleQ-2 is a *bona fide* FleQ binding site whose integrity is critical to FleQ activation of LapA transcription, although low level residual FleQ-dependent regulation may be supported by other sites.

## Discussion

The transcriptional organization of the *flhA-flhF-fleN-fliA* region was previously unresolved, as two separate studies proposed the transcriptional units *flhA* and *flhFfleNfliA* [47] or *flhAF* and *fleN-fliA* [46]. However, our RT-PCR results showing evidence of transcription across all three intergenic regions indicate that all four genes are co-transcribed as part of an operon (Fig 1). Furthermore, we have provided evidence that in addition to the main promoter located upstream from *flhA*, an internal promoter located within the *flhA* coding region and designated P*flhF* directs transcription of the three distal genes (Fig 2). While FleQ-independent transcription of *fliA* has been documented in *P*. *aeruginosa* [21], our RT-PCR results (Fig 1), along with the fact that a miniTn*5*-Km insertion in *flhF* results in a nonmotile, non-flagellated phenotype, while deletion of *flhF*, *fleN* or both does not prevent motility (Fig 3) strongly suggest that *fliA* transcription in *P*. *putida* is strictly dependent on the upstream P*flhA* and P*flhF* promoters and the phenotype of the insertion mutant is due to transcriptional polarity on *fliA*. The fact that readthrough transcription was detected at the *fliA-cheY* intergenic region in the wild-type, Δ*flhF*, Δ*fleN* and Δ*flhF-fleN* strains but not in the *flhF*::miniTn*5*-Km mutant indicates that transcription from P*flhA* and P*flhF* extends beyond *fliA* into *cheY*. This is in sharp contrast with the previous suggestion that *cheY* transcription is FliA-dependent [46]. We note however that such evidence was obtained from transcriptomics analysis performed with a *fliA*::Km insertion mutant, and therefore the lack of *cheY* transcription may be explained by transcriptional polarity of the insertion, as shown above for the *flhF*::miniTn*5*-Km mutant. In this regard, bioinformatics analysis failed to reveal significant matches to the FliA-RNA polymerase binding consensus at the *fliA-cheY* intergenic region, and a transcriptional fusion encompassing 263 bp of proximal *cheY* sequence, the 106 bp intergenic region and 289 bp of distal *fliA* sequence failed to reveal any significant FliA-regulated transcription (Antonio Leal-Morales, unpublished results). Given the short length of the subsequent *cheY*-*cheZ* (11 bp), *cheZ-cheA* (21 bp), *cheA-cheB* (47 bp), *cheB-motC* (0 bp) and *motC-motD* (2 bp) intergenic regions and the lack of predicted terminator or promoter sequences in them, we consider it likely that the P*flhA-* and P*flhF*-initiated transcriptional units extend across this region to encompass these six additional chemotaxis and flagellar motor genes.

Expression analysis revealed that the P*flhA* and P*flhF* are positively regulated by FleQ and negatively regulated by FleN, while FliA has no effect on either of the promoters (Fig 2). Epistasis analysis strongly suggests that FleN regulation is dependent on FleQ function, consistent with the proposed role of FleN as an antagonist for FleQ-dependent activation [43,44]. P*flhA* was previously shown to be a σ^N^-dependent Class II promoter [23]. A σ^N^-dependent promoter was previously described upstream from *flhF* in *P*. *putida* ATCC 12633 [47]. Although we cannot formally exclude that FleQ- and FleN-dependent regulation of the P*flhF* promoter is indirect, we consider it likely that P*flhF* is also a Class II promoter. Consistently with our observations, FleQ binding to genomic regions consistent with the location of the P*flhA* and P*flhF* promoters described here [54]. It is worth noting that P*flhF* displays considerable FleQ-independent basal expression (Fig 2). This is consistent with the presence of additional weak σ^70^-dependent promoters in this region, as previously shown [47]. As noted above, *fliA* lacks a promoter of its own, and should therefore be considered a Class II flagellar gene, unlike the Class I classification in *P*. *aeruginosa* [41]. FleQ-dependent regulation of FliA transcription has been documented in *Legionella pneumophila* and *Pseudomonas syringae* pv tomato [55,56].

While regulated flagellar location involving 'landmark' proteins has been documented for multiple bacterial species showing all types of flagellation patterns, a stochastic nucleation pattern leading to random flagellar localization has been proposed for *E*. *coli* and *Salmonella* [42,57]. In a *P*. *putida* Δ*flhF* mutant, the polar tuft of 3–4 flagella found in the wild-type was replaced by 1–2 single, randomly located flagella (Fig 3C). These results confirm a role for FlhF in determining the polar location of flagella, as shown previously in *P*. *putida* [47] and other organisms [32–37]. We propose that, in the absence of the 'landmark' protein FlhF, *P*. *putida* is still able to assemble flagella, albeit less efficiently (hence the small number of flagella) by means of stochastic nucleation, leading to randomly located flagella that still retain partial functionality. As a likely consequence of this aberrant flagellation pattern, smooth swimming was sometimes substituted by rolling diagonally or perpendicularly to the cell's long axis, a pattern that was exacerbated when *flhF* and *fleN* deletions were combined (S1 Fig, S1–S4 Movies). This motility behavior is reminiscent of tumbling motility typical of *Listeria*, a genus of Gram-positive bacteria bearing 1 to 5 peritrichous flagella [58]. Interestingly, the Δ*flhF* mutant displayed a relatively modest defect in swimming but was unable to swarm (Fig 3B and 3C), suggesting that polar flagellation is not a requisite for flagellar motility, but it is required for coordination of cell populations during swarming.

Similarly, we have confirmed a role of FleN in determining the number of flagella in *P*. *putida*. Strains lacking FleN displayed more flagella (Fig 3C) and faster swimming than their FleN^+^ counterparts, with smooth, rarely interrupted trajectories that often bent clockwise. Interestingly, changes in the number of flagella did not significantly influence the swimming speed and frequency of tumbles in *E*. *coli* [57,59], while longer run times with high directional persistence correlated with smaller numbers of flagella in *Bacillus subtilis* [60]. The contrast between these results and our observation suggest that the increase of swimming speed with the flagellar numbers may be a trait specific of polarly flagellated bacteria. On the other hand, bent swimming trajectories in flagellar motility are the result of a hydrodynamic effect due to wall-induced torque occurring in the presence of a nearby solid surface [61]. *P*. *putida* flagella can propel the cells in the forward (pushing) or in the backwards direction (pulling). These two forms of motility are distinguished by their tendency to clockwise- or counterclockwise-bent trajectories, respectively [62]. Accordingly, our results suggest that *fleN* inactivation results in a bias towards forward (pushing) motility, that may result in the observed defects in the swimming and swarming assays.

**Fig 9:**
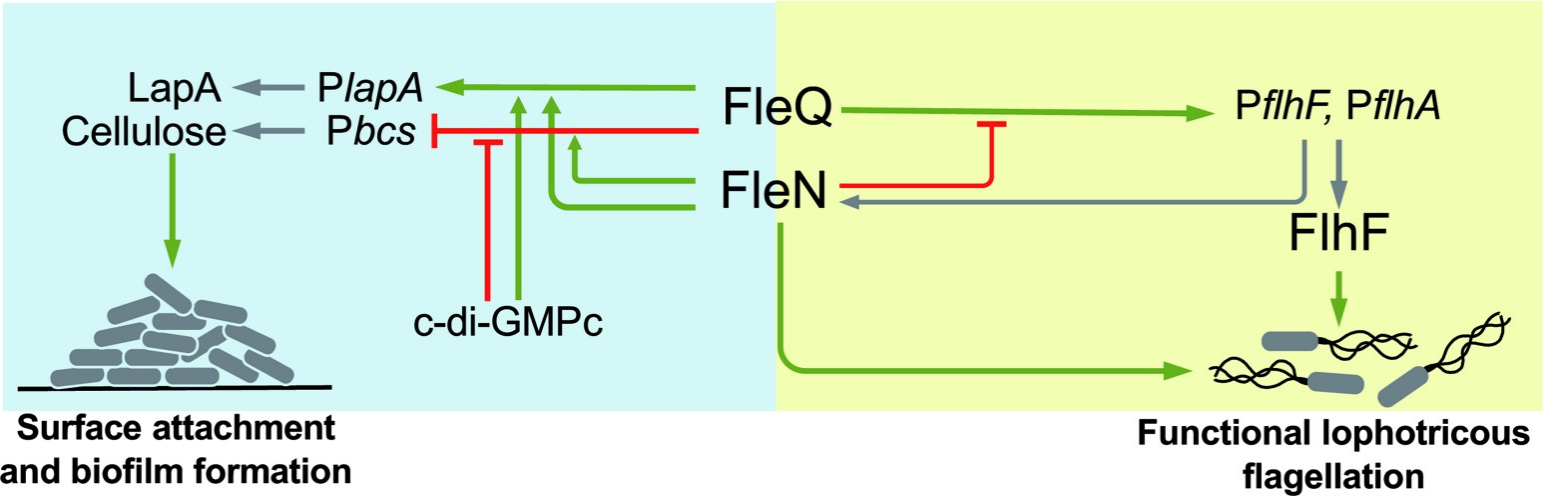
Model of the role of FleN in the planktonic-to-biofilm lifestyle switch. Green arrows denote positive effects. Capped red lines denote negative effects. Grey arrows connect promoters with end-products.

Our results position FleN as a major player in the transition between the planktonic and sessile lifestyles (Fig 9). Firstly, FleN antagonizes FleQ-dependent activation of the P*flhA* and P*flhF* promoters of the *flhAFfleNfliA* operon, encoding the regulatory elements relevant to flagellar biogenesis FlhF, FleN and FliA, as well as the flagellar secretion protein FlhA (Fig 2); secondly, FleN is required for normal surface attachment and biofilm formation (Fig 5); and finally, FleN is directly involved, along with FleQ and c-di-GMP, in the transcriptional regulation of the P*lapA* promoter, driving the synthesis of a high molecular weight adhesin, and the P*bcsD* promoter, responsible for transcription of the *bcs* cluster, encoding the cellulose synthase complex (Fig 6A).

We have found that FleN acts synergistically with FleQ to regulate transcription of *lapA* and the *bcs* cluster. In the case of the P*bcsD* promoter, FleN promotes FleQ repression *in vivo* at low c-di-GMP concentrations (Fig 6C). Such repression is released by an increase of intracellular c-di-GMP levels. Accordingly, FleN promotes FleQ interaction with the P*bcsD* promoter region *in vitro* and c-di-GMP antagonizes such effect (Fig 7B). Similar binding assays performed with a different P*bcsD* promoter fragment were recently reported [25], with equivalent results. Remarkably, the fragments used in both assays have an overlap region of only 41 bp (-61 to -21) relative to the annotated start codon. This region contains a putative FleQ binding site centered at position -34.5, matching 12 out of the 14 positions of the proposed consensus [53]. A putative σ^70^-type promoter overlaps this motif, thus providing a sound rationale for P*bcsD* promoter repression by blocking access of RNA polymerase to the promoter region [63,64]. A second, less conserved putative FleQ binding site is centered at position -74.5 and may contribute to the observed FleQ binding and repression observed here and previously [25,53], but further evidence is lacking.

Our results revealed that FleN acts synergistically with FleQ in the activation of *lapA* transcription *in vivo* at high c-di-GMP levels, but limits FleQ-dependent activation at a low c-di-GMP regime. Interestingly, c-di-GMP stimulated *lapA* transcription in the presence of FleN, but antagonized FleQ activation in its absence (Fig 6B). FleQ binds *in vitro* to two different P*lapA* fragments. In both cases, FleN stimulated FleQ-DNA interaction, which was increased further by the addition of c-di-GMP (Fig 7C and 7D). Similar results were recently obtained with P*lapA* probes largely overlapping the P*lapA*-2 fragment shown here [25]. Analysis of the interaction of FleQ with FleN and c-di-GMP at the P*lapA* promoter region and its functional consequences is complicated by the presence of six functional *lapA* promoters, up to four putative FleQ binding sites and six binding sites for an additional transcription factor, Fis, which is also a positive regulator of *lapA* transcription [52]. Our P*lapA*-2 probe contains two putative FleQ binding sites, as well as four promoters, including the strong P*lapA*3 promoter [52,53], suggesting that this region is likely relevant to the regulation. While our site-directed mutagenesis results revealed that a single site (FleQ-2) is critical for most of the FleQ-dependent regulation observed (Fig 8), the presence of multiple, closely clustered FleQ binding sites may be relevant to the ability of FleN to modulate the effect of c-di-GMP on FleQ-dependent activation, as previously observed in the regulation of the *P*. *aeruginosa pel* promoter [65]. On the other hand, our P*lapA*-2 probe contains two weak *lapA* promoters and a putative FleQ binding motif. This fragment is located upstream from the fragments recently assessed by Nie *et al*. (2017) [25], and therefore FleQ-DNA interaction in this region was not previously documented. The similar behavior in the interaction of FleQ with FleN and c-di-GMP in the two promoter fragments suggests a complex coordinated regulatory mechanism involving several FleQ binding sites and multiple promoters may be involved in the regulation of *lapA* transcription.

## Materials and methods

### Bacterial strains and growth conditions

Bacterial strains used in this work are summarized in Table 1. Planktonic cultures of *E*. *coli* and *P*. *putida* strains were routinely grown in Luria-Bertani (LB) broth [66] at 37°C and 30°C, respectively, with 180 rpm shaking. For solid media, Bacto-Agar (Difco) was added to a final concentration of 18 g l^-1^. Antibiotics and other additions were used, when required, at the following concentrations: ampicillin (100 mg l^-1^), carbenicillin (0.5 g l^-1^), kanamycin (25 mg l^-1^), rifampicin (10 mg l^-1^), chloramphenicol (15 mg l^-1^), gentamycin (10 mg l^-1^), tetracycline (5 mg l^-1^), 5-bromo-4-chloro-3-indoyl-β-D-galactopyranoside (X-gal) (25 mg l-1) and sodium salicylate (2 mM). All reagents were purchased from Sigma-Aldrich, except for X- Gal, which was purchased from Fermentas.

**Table 1:** Bacterial strains, plasmids and oligonucleotides used in this work. Underlined bases indicate oligonucleotide positions that differ from the corresponding templates.

**Bacterial strain**	**Genotype/phenotype**	**Reference/source**
***E*. *coli***		
DH5α	Ψ80d*lacZ*ΔM15 Δ(*lacZYA-argF*)U169 *recA*1 *endA*1 *hsdR*17 (r_k_^-^ m_k_^+^) *supE*44 *thi*-1 *gyrA relA*1	[71]
***P*. *putida***		
KT2440	mt-2 *hsdR*1 (r^-^ m^+^)	[72]
KT2442	mt-2 *hsdR*1 (r^-^ m^+^) Rif^r^	[72]
KT2440 *fliA*::*aphA-3*	KT2440 *fliA*::Km. Km^r^	[46]
MRB49	KT2442 *flhF*::miniTn*5*-Km. Rif^r^ Km^r^	[45]
MRB52	KT2442 Δ*fleQ*	This work
MRB69	KT2442 Δ*flhF*	This work
MRB71	KT2442 Δ*fleN*	This work
MRB78	KT2442 Δ*flhF-fleN*	This work
MRB101	KT2442 Δ*fleN* Δ*fleQ*	This work
**Plasmid**	**Genotype/phenotype**	**Reference/source**
pENTR/D-TOPO	Vector for directional TOPO cloning. Km^r^	Thermo Fisher Scientific
pEX18-Tc	Gene replacement vector with MCS from pUC18. Tc^r^ Sac^s^	[73]
pMPO284	pPS854-derived vector containing pUTminiTn*5*Km Km^r^ gene flanked by the FRT sites, Ap^r^ Km^r^	[26]
pMRB1	pBBR1-MCS4-derived broad host-range *gfp*mut3::*lac*Z transcriptional fusion vector. Ap^r^	[26]
pMRB2	pBBR1-MCS4-derived vector containing the Gateway conversion cassette *attR*1-Cm^r^-*ccd*B-*attR*2. Ap^r^ Cm^r^	[26]
pMRB3	pBBR1-MCS4-derived vector containing the Gateway conversion cassette *attR*1-Cm^r^-*ccd*B-*attR*2. Ap^r^ Cm^r^	[23]
pMRB67	pMRB2-derived vector containing a P*lapA-gfp*mut3-*lac*Z transcriptional fusion. Ap^r^	[23]
pMRB89	Expression vector for *E*. *coli* YhjH containing *nahR*-P*sal*-T*nasF*-*yhjH*. Str^r^	[26]
pMRB95	pTYB12-based vector for FleQ overproduction. Ap^r^	This work
pMRB96	pEX18-Tc with Km^r^ gene flanked by FRT sites cloned between *fleQ* flanking regions. Tc^r^ Km^r^ Sac^s^	This work
pMRB104	pEX18-Tc with Km^r^ gene flanked by FRT sites cloned between *flhF* flanking regions. Tc^r^ Km^r^ Sac^s^	This work
pMRB105	pEX18-Tc with Km^r^ gene flanked by FRT sites cloned between *fleN* flanking regions. Tc^r^ Km^r^ Sac^s^	This work
pMRB112	pMRB2-derived vector containing a P*bcsD-gfp*mut3-*lac*Z transcriptional fusion. Ap^r^	[23]
pMRB115	pMRB3-derived vector containing a P*flhA-gfp*mut3-*lac*Z transcriptional fusion. Ap^r^	[23]
pMRB132	pTYB12-based vector for FleN overproduction. Ap^r^	This work
pMRB144	pEX18-Tc with Km^r^ gene flanked by FRT sites cloned between *flhF-fleN* flanking regions. Tc^r^ Km^r^ Sac^s^	This work
pMRB158	pMRB3-derived vector containing a P*flhF-gfp*mut3-*lac*Z transcriptional fusion. Ap^r^	This work
pRK2013	Helper plasmid for triparental mating. ColE1 replicon. Km^r^	[74]
pTYB12	Expression vector for N-terminal Intein–chitin-binding domain protein fusions. Apr	New England Biolabs
pYedQ	pRK404A-derived plasmid expressing E. coli YedQ from the P*lac* promoter. Tc^r^	[75].
**Oligonucleotide**	**Sequence (5’ to 3’)**	
flhF-up-Fwd	ACCCGAATTCCGAACTGGCGATCAACCC	
flhF-up-Rev	CGCAGGATCCGCATTATCCCCTACCTCA	
flhF-down-Fwd	CGGCGGATCCGTTGACCATGAAGCGTGT	
flhF-down-Rev	TGCAAAGCTTGCTCGACAAAGAACTCCA	
fleN-up-Fwd	CCTGGAATTCCTGCTGGAAGTGCAACTC	
fleN-up-Rev	TGCAGGATCCCCATGTCTGTTCTTTACC	
fleN-down-Fwd	GGACGGATCCTATGAACGCCAGCGGCTT	
fleN-down-Rev:	TTTTAAGCTTCTTGTCCAATTAGACCTC	
flhA-RT-fwd	TCTTCACGTTCAACATCGCC	
flhA-RT-rev	TCCATCGAGCCGTAGAACTC	
Pint_FlhA_Fwd	CACCCTCTGAAGACATGGGCAAG	
Pint_FlhA_Rev2	CACGGACCAGTTTCATGGCC	
flhA-flhF-RT-fwd	CTTCTGGAACCTAGCATGGC	
flhA-flhF-RT-rev	AATGCGCGTGTGGGTCTT	
flhF-miniTn5DST2-fwd	CATGCAACTGGAAACCTTGG	
flhF-miniTn5DST2-rev	GCCGTGGGTTGTGATAGAGA	
flhF-fleN-RT-fwd	GAGCCTTGCCATCAGTCATG	
flhF-fleN-RT-rev	CAGCAACACGTCGACATTGG	
fleN-fliA-RT-fwd	GACCGCTTCCTTGACGTTG	
fleN-fliA-RT-rev	CGTCGTATTTGTTGGCCACT	
fliA-cheY-RT-fwd	CAAGGAAATCGGTGAGGTGC	
fliA-cheY-RT-rev	CCGCTTCAATGATCTGGTCG	
PflhF-fwd	CACCCTCTGAAGACATGGGCAAG	
PflhF-rev	CACGGACCAGTTTCATGGCC	
fleN-fwd-NdeI	AACACATATGGGTAGCATGCATCCC	
fleN-rev-EcoRI	CCGCGAATTCTCATAGTACGGGTCCCGC	
PlapA1-fwd-HindIII	TGACAAGCTTCGACTGACTTCGGATTCC	
PlapA1-rev	AGCTATGTGTCGCAAATCAA	
PlapA2-fwd	TTGATTTGCGACACATAGCT	
PlapA2-rev-BamHI	GCCCGGATCCATTGGACTCTCCGTGTGACC	
PbcsD-fwd-HindIII	CCCAAGCTTGTTGATCGCCAGCACCTGG	
PbcsD-rev	GACTCATGTCAAAAAAACGACAAAAATGA	
lapA-qRT-fwd	AGCATTGTCGGCCAGGTTATT	
lapA-qRT-rev	TCGATAAGTACACGCCGGATG	
bcsD-qRT-fwd	CATTCCGCCTTCAAACCGTTT	
bcsD-qRT-rev	GGAAATCGCCAACAACTGCAT	

### Plasmid and strain construction

Plasmids and oligonucleotides used in this work are summarized in Table 1. All DNA manipulations were performed following standard protocols [66]. Restriction and modification enzymes were used according to the manufacturers' instructions (Fermentas, Roche and NEB). When required, blunt ends were generated using the Klenow fragment or T4 DNA polymerase. *E*. *coli* DH5α was used as a host in cloning procedures. All cloning steps involving PCR were verified by commercial sequencing (Secugen). Plasmid DNA was transferred to *E*. *coli* and *P*. *putida* strains by transformation [67], triparental mating [68] or electroporation [69]. Site-specific integration of miniTn*7* derivatives in *P*. *putida* strains was performed essentially as described [70].

To construct a *P*. *putida* KT2442 derivative with an in-frame deletion of the *flhF* gene (MRB69), 811 bp and 894 bp from the upstream and downstream chromosomal regions flanking *flhF* were PCR-amplified with oligonucleotide pairs FlhF-up-Fw/FlhF-up-Rev (upstream region) and FleF-dw-Fwd/FlhF-dw-Rev (downstream region). The PCR products were cleaved with EcoRI and BamHI or BamHI and HindIII, respectively, and three-way ligated into EcoRI- and HindIII- digested pEX18Tc. A BamHI-excised FRT-flanked kanamycin resistance cassette from pMPO284 was then cloned into the BamHI site, yielding pMRB104. This plasmid was transferred to *P*. *putida* KT2442 by electroporation. Selection of integration, allelic replacement and FLP-mediated excision of the kanamycin resistance marker was performed essentially as described [73,76]. This strategy led to the complete deletion of *flhF* minus its start and stop codons and its replacement with a short ORF spanning the start codon, the single FRT scar and the stop codon. Δ*fleQ*, Δ*fleN* and Δ*flhF-fleN* mutants were generated using an equivalent approach. Plasmids containing 800–900 pb upstream and downstream regions flanking the target genes and the FRT-flanked kanamycin resistance cassette were constructed. These plasmids, named pMRB96, pMPRB105 and pMRB144, were transferred by electroporation to KT2442 to generate MRB52 (Δ*fleQ*), MRB71 (Δ*fleN*) and MRB78 (Δ*flhF-fleN*). A Δ*fleQ*Δ*fleN* mutant (MRB101) was also constructed by using pMRB105 to electroporate MRB53 (Δ*fleQ*). The structure of the deleted loci was verified by PCR and Southern blot.

For the construction of pMRB132, a plasmid overproducing a chitin-binding domain (CBD)- intein-FleN fusion for protein purification, the *fleN* open reading frame was PCR amplified with FleN_Fwd_NdeI/FleN_Rev_EcoRI primers and cloned into NdeI- and EcoRI-cleaved pTYB12 (New England Biolabs).

A 1381 pb fragment containing the putative P*flhF* promoter region (positions -1323 pb to +57 relative to the *flhF* start codon) was amplified using oligonucleotides Pint_FlhA_Fwd and Pint_FlhA_Rev as primers, and inserted in the directional TOPO cloning vector pENTR^TM^/D-TOPO. This region was subsequently transferred using the Gateway recombination technology (Thermo Fisher Scientific) into the Gateway *gfp*mut3-*lacZ* fusion vector pMRB2, to produce pMRB158.

### Biofilm growth and quantification

For most procedures involving biofilm growth, overnight cultures grown in LB or K10T-1 broth [77] were diluted in the same medium to an A_600_ of 0.1 and 150 μl were dispensed into wells of Costar 96 microtiter polystyrene plates (Corning). The plates were incubated at 25°C with moderate shaking (150 rpm) for the desired period of time and processed for planktonic and biofilm growth quantification, essentially as described [78]. Serial dilution-based growth curves were performed as described [45]. For each experiment, at least 3 biological replicates were assayed in octuplicate.

For pellicle and aggregate detection, fresh colonies were inoculated in glass tubes containing 5 ml of K10T-1 broth. Cultures were incubated overnight at 30°C with shaking, after which the tubes were placed on a rack for 10 minutes and documented by digital photography.

### Flagellar motility (swimming and swarming) assays

Swimming assays were adapted from [79]. Tryptone motility plates containing 0.3% Bacto-agar (Difco) were toothpick-inoculated with fresh colonies and incubated for 12 h at 30°C. Digital photographs were taken, and the swimming zone diameter was measured and normalized that of the wild-type. At least 3 biological replicates were assayed for each strain.

Swarming assays were performed essentially as described by [80], with some modifications. Two and a half microliters of overnight LB cultures were spotted onto the center of plates containing 0.5% PG-agar [proteose peptone No. 3 (Difco 212693), 0.5%, and glucose, 0.2%, with Difco Bacto-Agar] and plates were incubated at 25ºC for 40 hours. At least two biological replicates were assayed in three separate experiments.

### Microscopy techniques

Phase contrast microscopy of surface adhesion was performed on a Leica DMI4000B inverted microscope using 20x objective and 1.6x ocular magnification. Cells from mid-exponential (A_600_ = 0.2–0.3) LB cultures of the selected *P*. *putida* strains were serially diluted (10^2^−10^4^) in LB and samples were transferred to wells of Costar 96 microtiter polystyrene plates (Corning). Attachment was allowed to proceed for 30 minutes, planktonic cells were removed by washing twice with 150 μl LB, and then 50 μl LB was added to the wells. For short-term assessment of surface attachment, plates were incubated at room temperature for 3 hours, and then cells deposited on the plane of the well surface were video recorded for 1 minute.

The Leifson flagellum staining procedure for light microscopy was carried out according to Clark [81], with some modifications. A 1.2% (w/v) solution of basic fuchsine prepared in 95% (v/v) ethanol and left overnight at 25°C was mixed with an equal volume of a solution of 0.75% NaCl (w/v) and 1.5% (w/v) tannic acid prepared in double-deionized water. The final pH of the dye was adjusted with 1 N NaOH to pH 5.0. Swarmer cells were resuspended in 10 mM MgCl_2_, adjusted to an OD_600_ of 1.0, and fixed by adding 1 ml of 4% (v/v) formaldehyde solution per ml of culture for 20 min. The suspension then was centrifuged, washed with double-deionized water, and resus- pended in 1 ml of double-deionized water. The slides were cleaned by soaking them for 24 h at room temperature in acid dichromate solution, rinsed with double-deionized water, and then air dried. A large drop of culture suspension was deposited on the centre of the slide and was allowed to run down its length and then air dried. Subsequently, 1 ml of dye solution was added and kept for 40 min, and the slide was washed with tap water, air dried, mounted with Merko-glass, and examined under a Zeiss Axioskop microscope.

### β-galactosidase assays

For β-galactosidase assays of *lacZ* fusions, overnight cultures bearing the corresponding fusion plasmids were diluted in 5 ml LB to an A_600_ of 0.01 and incubated for 24 hours at 30°C with shaking. Growth was then stopped, and β-galactosidase activity was determined from sodium dodecyl sulfate- and chloroform-permeabilized cells as described [82].

### RNA preparation and RT-PCR

Total RNA was prepared from stationary phase cultures essentially as described [83]. Reverse transcription (RT) of total RNA (5 μg) was carried out using the high-capacity cDNA Archive kit (Applied Biosystems), with random hexamers as primers. The primer pairs flhA-RT-fwd/ flhA-RT-rev, flhA-flhF-RT-fwd/ fleN-fliA-RT-fwd, flhF-miniTn5DST2-fwd/flhF-miniTn5DST2-rev, flhF-fleN-RT-fwd/flhF-fleN-RT-rev, fleN-fliA-RT-fwd /fleN-fliA-RT-rev and fliA-cheY-RT-fwd/fliA-cheY-RT-rev was used to amplify fragments of 457 pb, 452 pb, 359 pb, 454 pb, 432 pb and 496 pb, respectively. Reactions were performed with 50 ng cDNA as template in a 25-cycle PCR program. Negative and positive controls were performed with total RNA or genomic DNA as templates, respectively. The RT-PCR products obtained were resolved by 1% agarose gel electrophoresis and visualized by ethidium bromide staining.

### Protein purification and gel mobility shift assays

FleQ and FleN were overproduced from the T7 promoter as a N-terminal intein-CBD fusion from the overproducing strain NCM631 harbouring pMRB95 or pMRB132 and pIZ227 by using the IMPACT kit from NEB (#E6901S Beverly, MA) as previously described for FleQ [23]. A summary of FleN overproduction an purification procedure is shown in S2 Fig. Protein concentration was calculated using the Protein Assay kit from Bio-Rad, according to the manufacturer’s protocol, and process efficiency and purity was estimated by SDS-PAGE.

Gel mobility shift assays were performed essentially as described [44]. The PlapA-1, PlapA-2 and PbcsD fragments were PCR amplified from genomic DNA and the products were cleaved with HindIII and gel purified. DNA fragments were labelled by filling in 5′ over-hanging ends using the Klenow fragment in a reaction mixture containing [α-^32^P]-dCTP. Unincorporated nucleotides were removed using the MSB Spin PCRapace kit (Invitek). FleQ-DNA complexes were formed at room temperature for 30 min in 10 μl reaction containing 10 ng/μl labelled-probe, 0.2 μg/μl poly-dI-dC and increasing amounts of purified FleQ in binding buffer (10 mM Tris-HCl [pH 7.8], 50 mM KCl, 8 mM magnesium acetate, 50 ng/l BSA, 5% glycerol). When required, 50 μM ATP, 300 μM c-di-GMP or different concentrations of FleN were added to the binding reactions. Reactions were stopped with 3 μl of loading buffer (0.125% w/v bromophenol blue, 0.125% w/v xylene cyanol, 10 mM Tris HCl (pH 8), 1 mM EDTA, 30% glycerol) and samples were separated on a 5% polyacrylamide native gel in TGE buffer (10 mM Tris [pH 8.8], 380 mM glycine, 1 mM EDTA) at 4°C. Dried gels were exposed on a phosphoscreen and were visualized with a Typhoon 9410 scanner and analysed using the ImageQuant software (Amersham).

### ATPase activity

FleN ATPase activity was assayed by measuring the inorganic phosphate production using the Enzchek Phosphate Assay Kit (Molecular Probes). Reactions were set up essentially as previously described [43]. FleN was added to the reaction mixtures at 1.3 μM and incubated for 30 min at room temperature before 2 mM ATP was added. A_360_ was measured after 3 h and used to calculate the specific activity.

## Supporting information

S1 Fig Flagellation patterns of the *flhF* and *fleN* mutants. Flagellar stain of the wild-type KT2442, the Δ*flhF* mutant MRB69, the Δ*fleN* mutant MRB71 and the Δ*flhF-fleN* mutant MRB78 strains.
(PDF)

S2 Fig Flagellar motility tracks of wild-type and mutant *P*. *putida* strains. Five cells from each video sequence of the wild-type, Δ*flhF*, Δ*fleN* and Δ*flhF-fleN* strains were monitored over time. Tracks were generated using the MtrackJ plugin of ImageJ.
(PDF)

S3 Fig SDS-PAGE gel showing FleN overproduction and purification. Lane 1: uninduced overproducing strain, whole cells; lane 2: induced overproducing strain, whole cells; lane 3: induced overproducing strain, clarified soluble extract; lane 4: chitin affinity resin flow-through; lane 5: wash buffer eluate; lane 6: chitin-bound protein prior to cleavage; lane 7: DTT wash solution prior to incubation; lane 8: chitin-bound protein after cleavage; lane 9: eluted protein after DTT incubation. M: molecular weight marker (sizes in kDa)
(PDF)

S4 Fig Predicted FleQ-binding sites at the P*lapA* and P*bcsD* promoter regions. (PDF)

S5 Fig Gel mobility shift assays on the PbcsD and PlapA promoters. Panels A, B and C: Autoradiograph of a representative PAGE gel containing the indicated probe, FleQ and/or FleN at the indicated concentrations. Assays were performed in the absence (-) or in the presence (+) of c-di-GMP and in the absence (-) or in the presence (+) of ATP. Closed arrowheads denote the free DNA probes and open arrowheads denote the retarded complexes.
(PDF)

S1 Movie Near surface flagellar motility of the wild-type strain KT2442. (MOV)

S2 Movie Near surface flagellar motility of the Δ*flh*F mutant. (MOV)

S3 Movie Near surface flagellar motility of the Δ*fleN* mutant. (MOV)

S4 Movie Near surface flagellar motility of the Δ*flh*F-*fleN* mutant. (MOV)
