# Supplementary figures and images for: Transcriptional organization, regulation and functional analysis of flhF and fleN in Pseudomonas putida
Source: PLoS One. 2019 Mar 19;14(3):e0214166. doi: 10.1371/journal.pone.0214166 (PMC6424431; doi:10.1371/journal.pone.0214166)

**Wild-type**

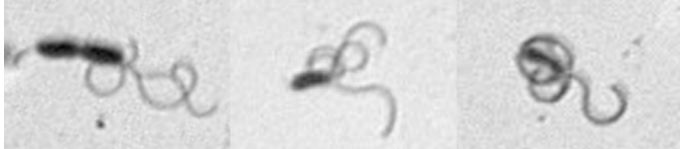

**$\Delta flhF$**

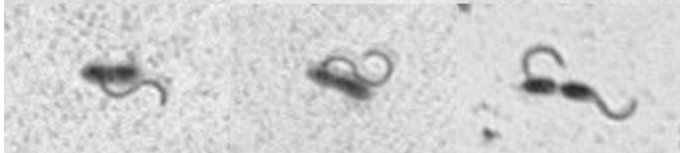

**$\Delta fleN$**

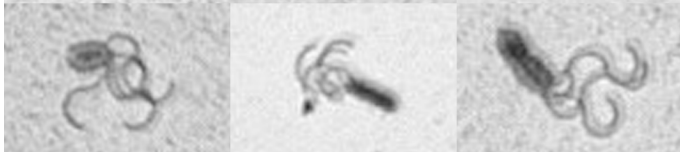

**$\Delta flhF-fleN$**

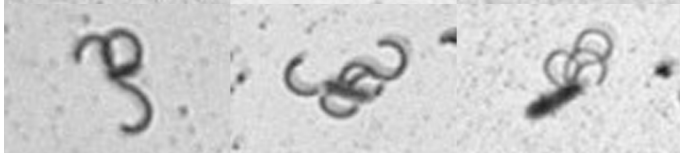

Supplement: S1 Fig — Flagellar stain of the wild-type KT2442, the ΔflhF mutant MRB69, the ΔfleN mutant MRB71 and the ΔflhF-fleN mutant MRB78 strains. (PDF) [file pone.0214166.s001.pdf]

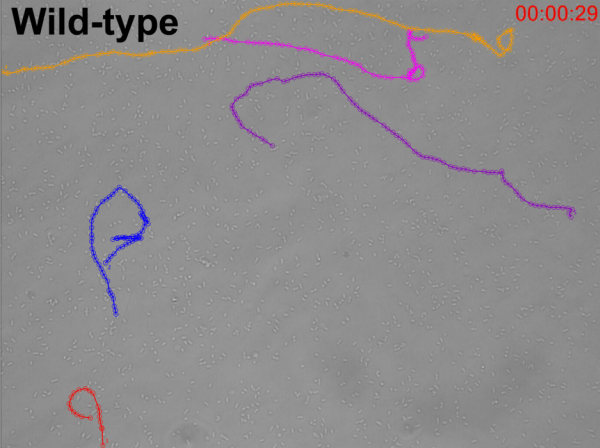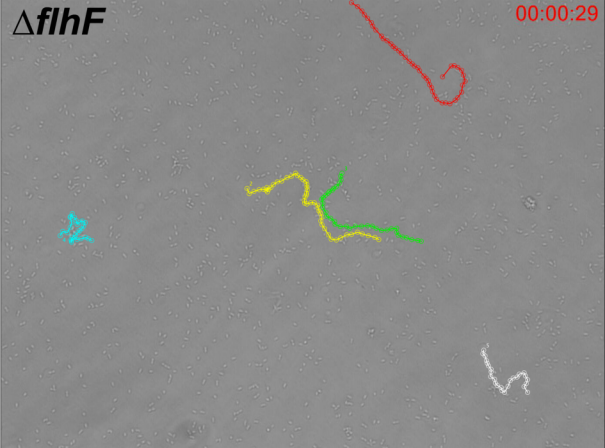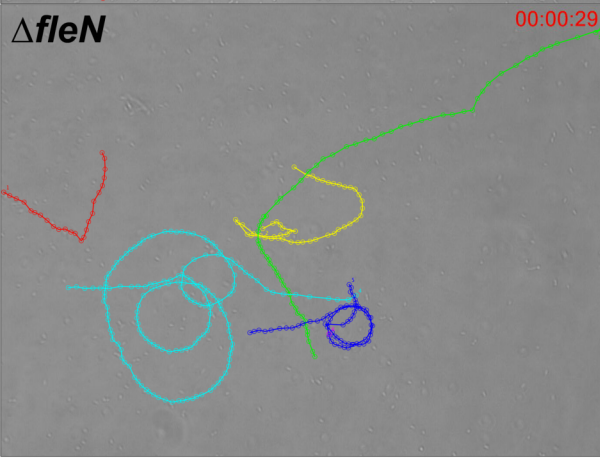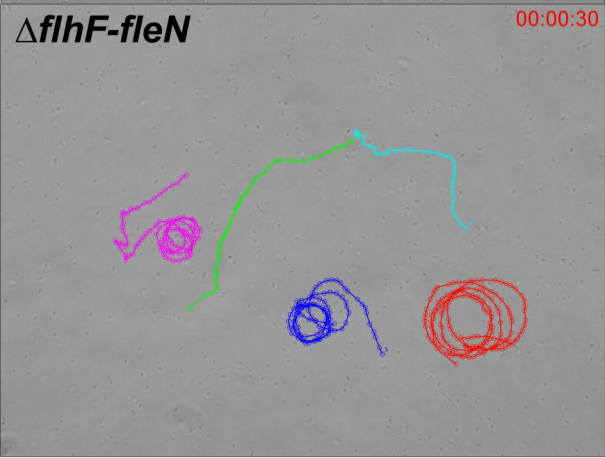

Supplement: S2 Fig — Five cells from each video sequence of the wild-type, ΔflhF, ΔfleN and ΔflhF-fleN strains were monitored over time. Tracks were generated using the MtrackJ plugin of ImageJ. (PDF) [file pone.0214166.s002.pdf]

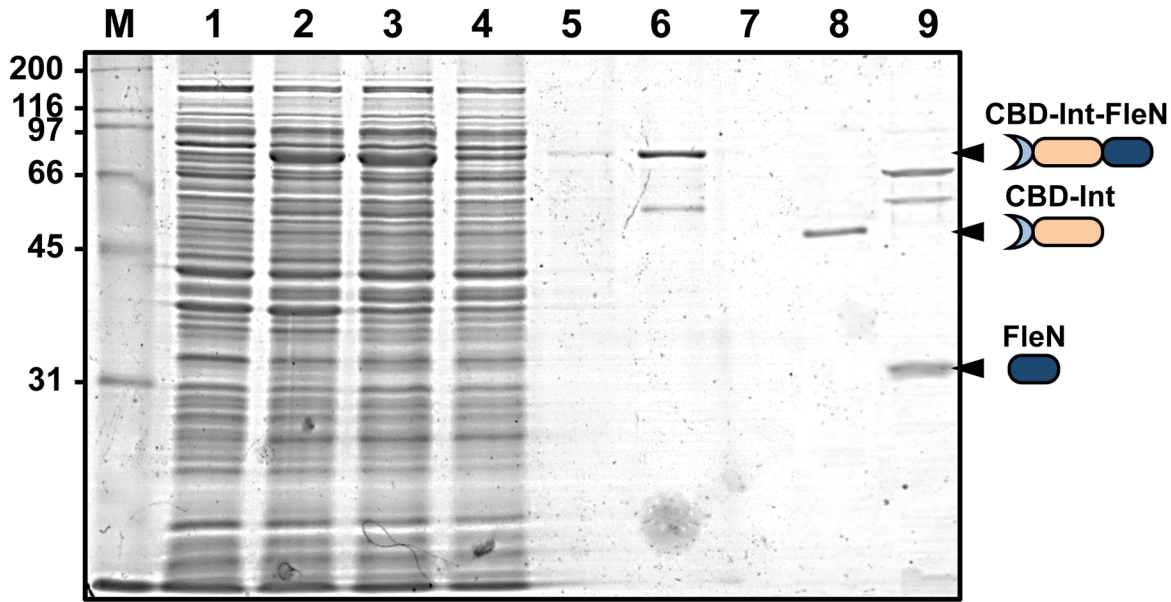

Supplement: S3 Fig — Lane 1: uninduced overproducing strain, whole cells; lane 2: induced overproducing strain, whole cells; lane 3: induced overproducing strain, clarified soluble extract; lane 4: chitin affinity resin flow-through; lane 5: wash buffer eluate; lane 6: chitin-bound protein prior to cleavage; lane 7: DTT wash solution prior to incubation; lane 8: chitin-bound protein after cleavage; lane 9: eluted protein after DTT incubation. M: molecular weight marker (sizes in kDa) (PDF) [file pone.0214166.s003.pdf]

**A**

|                 |   |   |   |   |   |   |   |   |
|-----------------|---|---|---|---|---|---|---|---|
| FleQ ( $\mu$ M) | - | - | - | - | 2 | 2 | 2 | 2 |
| FleN ( $\mu$ M) | - | - | 2 | 2 | - | - | 2 | 2 |
| c-di-GMP        | - | + | - | + | - | + | - | + |
| ATP             | + | + | + | + | + | + | + | + |

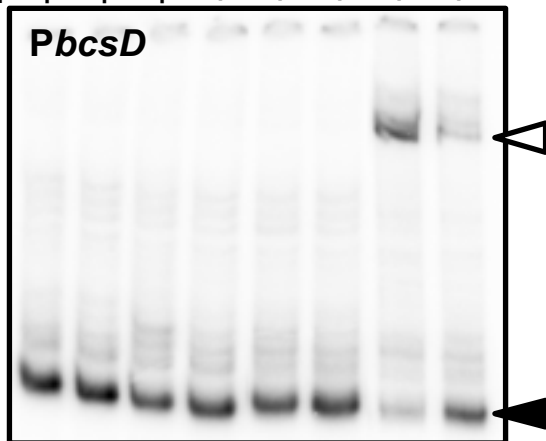**B**

|                 |   |   |     |   |     |   |
|-----------------|---|---|-----|---|-----|---|
| FleQ ( $\mu$ M) | - | - | 0.5 | 2 | 0.5 | 2 |
| FleN ( $\mu$ M) | - | 2 | -   | - | 0.5 | 2 |
| c-di-GMP        | - | - | -   | - | -   | - |
| ATP             | + | + | +   | + | +   | + |

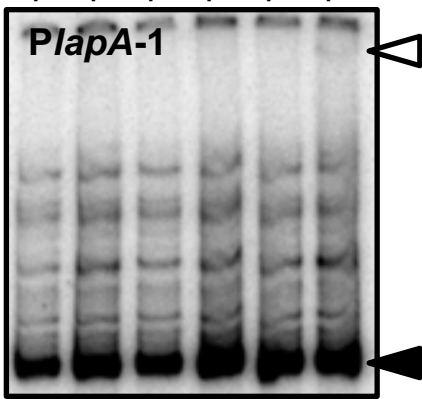**C**

|                 |   |   |     |   |     |   |
|-----------------|---|---|-----|---|-----|---|
| FleQ ( $\mu$ M) | - | - | 0.5 | 2 | 0.5 | 2 |
| FleN ( $\mu$ M) | - | 2 | -   | - | 0.5 | 2 |
| c-di-GMP        | - | - | -   | - | -   | - |
| ATP             | + | + | +   | + | +   | + |

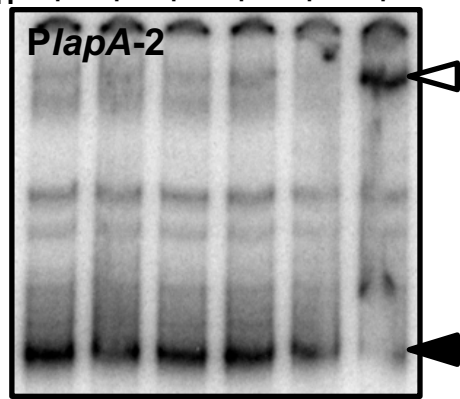

Supplement: S5 Fig — Panels A, B and C: Autoradiograph of a representative PAGE gel containing the indicated probe, FleQ and/or FleN at the indicated concentrations. Assays were performed in the absence (-) or in the presence (+) of c-di-GMP and in the absence (-) or in the presence (+) of ATP. Closed arrowheads denote the free DNA probes and open arrowheads denote the retarded complexes. (PDF) [file pone.0214166.s005.pdf]
