# Supplementary material for: Transcriptional organization, regulation and functional analysis of flhF and fleN in Pseudomonas putida
Source: PLoS One. 2019 Mar 19;14(3):e0214166. doi: 10.1371/journal.pone.0214166 (PMC6424431; doi:10.1371/journal.pone.0214166)
Supplement: S4 Fig — (PDF) [file pone.0214166.s004.pdf]

P*bcsD* fragment (-89 to -1)

FleQ-1

GTCAA**AAAATTGAC** **FleQ consensus**

GCCTTGAT**G**CTGT**ATAATTGAC**GCAACTTTGTGTTACGCATCATTTTT**GTC**GT**TTTTTTGAC**ATGAGTCATAAAGCGTCAGCAGAGGAT

FleQ-2

GTCAA**TTTTTTGAC** **FleQ consensus**

-35 **-10**

P*bcs*

P*lapA*-1 fragment (-680 to -592)

FleQ-1

GTCAAA**AAA**TT**G**AC **FleQ consensus**

AGCTTAGTCACT**GTCA**GT**AAA**CA**G**G**C**GCTGGTTTGTTGGCGCATATCTGTGGCTTGTTAGTCAAAATTCATCTATAGAGTGCGGATTCT

-35 **-10**

P*lapA8*

P*lapA*-2 fragment (-209 to -32)

FleQ-2

GTCAATTT**TTTG**AC **FleQ consensus**

GGTACAAATATCAATGTGACATTACATTGCCGATTGTTTAGGATGGCATGTATAAG**GTCAAT**AG**TTTG**G**C**AGTCAGGCAATTCCAAAAA

GACCC**T**AG**TTTG**GC **FleQ-2mut site**

-35 **-10**

P*lapA3*

FleQ-3

GT**C**AA**AA**A**ATTGAC** **FleQ consensus**

GTTATA**G**A**C**GG**AA**T**ATTGAC**GTCAAAAACGTCAAGAGATCATCGACATAGTTCCGCCTGAAGTGGCTAGCAAGCGCCGCTCTGGCAGGG

GA**C**GG**AA**T**A**GGTC**C** **FleQ-3mut site**
